# Oxygen-dependent metabolism of dissolved organic matter in hypoxic marine environments

**DOI:** 10.1093/ismejo/wrag220

**Published:** 2026-08-26

**Authors:** Benjamin N Daniels, Sarah Wolf, Qi Chen, Fabian Wittmers, Kayla Stanley, Clare Jayawickrama, Edward W Davis II, Abzer K Pakkir Shah, Craig A Carlson, Daniel Petras, Francis Chan, Stephen J Giovannoni

**Affiliations:** Department of Microbiology, Oregon State University, Corvallis, 1500 SW Jefferson Avenue, 97331 OR, United States; Department of Microbiology, Oregon State University, Corvallis, 1500 SW Jefferson Avenue, 97331 OR, United States; Department of Microbiology, Oregon State University, Corvallis, 1500 SW Jefferson Avenue, 97331 OR, United States; Department of Microbiology, Oregon State University, Corvallis, 1500 SW Jefferson Avenue, 97331 OR, United States; Department of Microbiology, Oregon State University, Corvallis, 1500 SW Jefferson Avenue, 97331 OR, United States; Department of Microbiology, Oregon State University, Corvallis, 1500 SW Jefferson Avenue, 97331 OR, United States; Center for Quantitative Life Sciences, Oregon State University, Corvallis, 1500 SW Jefferson Avenue, 97331 OR, United States; Department of Biochemistry, University of California, Riverside, 900 University Avenue, 92521 CA, United States; CMFI Cluster of Excellence, University of Tüebingen, Geschwister-Scholl-Platz, 72074 Tübingen, Germany; Department of Ecology, Evolution, and Marine Biology, University of California Santa Barbara, University of California, 93106 CA, United States; Department of Biochemistry, University of California, Riverside, 900 University Avenue, 92521 CA, United States; Department of Integrative Biology, Oregon State University, Corvallis, 1500 SW Jefferson Avenue, 97331 OR, United States; Department of Microbiology, Oregon State University, Corvallis, 1500 SW Jefferson Avenue, 97331 OR, United States

**Keywords:** hypoxia, dissolved organic matter, oxygen-dependent metabolism, oxygen minimum zones

## Abstract

Low-oxygen marine environments are dynamic hotspots where microbial activity and dissolved organic matter (DOM) cycling are altered, with implications for understanding ecosystem responses to intensifying anthropogenic pressures. The hypoxic barrier hypothesis (HBH) proposed a mechanism by which DOM cycling could be altered under hypoxia by the inhibition of non-respiratory oxidase enzymes, whereas respiration continued. To investigate oxygen-dependent changes in DOM composition and consequences of the HBH, we combined controlled mesocosm experiments with non-targeted metabolomics to identify classes of compounds that accumulate in hypoxic treatments. Many of these compounds require non-respiratory oxidase enzymes for catabolism in Kyoto Encyclopedia of Genes and Genomes (KEGG) metabolic maps, and when tested in experiments with microbial communities, their catabolism was inhibited by hypoxia, validating the concept of oxygen-dependent DOM (ODDOM). The identified ODDOM molecules displayed chemical characteristics associated with increased DOM stability, including increased aromaticity, carbon oxidation state, and decreased saturation. In a meta-analysis of Fourier transform ion cyclotron resonance mass spectrometry (FT-ICR-MS) datasets from three oceanographic cruises that included samples collected across oxygen gradients and within oxygen minimum zone (OMZ) waters, molecules with chemical properties similar to the mesocosm-derived ODDOM accumulated within the oxycline of the North Pacific Ocean. In the mesocosm experiments, the growth of many microbial taxa was unaffected by hypoxia, and others were either stimulated or inhibited by hypoxia, suggesting unexplored complexity in microbial adaptations to oxygen concentrations in this range. These findings suggest that hypoxia can mechanistically constrain enzymatic pathways determining DOM bioavailability, with implications for microbial carbon cycling and microbial evolution in expanding ocean OMZs.

## Introduction

Marine dissolved organic matter (DOM) is the largest pool of reduced carbon in the oceans, comparable to the magnitude of CO_2_ in the atmosphere [1, 2]. Free oxygen in the oceanic water column plays a key role in the remineralization of DOM, yet the dynamics of carbon turnover at molecular scales in low-oxygen environments remain poorly understood [3, 4]. Microbial carbon cycling regulates the size and composition of the DOM pool and ultimately shapes carbon sequestration through the microbial carbon pump, altering global biogeochemical cycles and climate feedback [1, 5]. Anthropogenic activities have intensified marine hypoxia, particularly in coastal regions, driving the expansion of oxygen minimum zones (OMZs) and underscoring the need to resolve the mechanisms controlling carbon storage in these environments [6, 7].

Previous studies have observed the accumulation of total organic carbon (TOC) in hypoxic systems, yet the mechanisms by which this TOC accumulates remain uncertain [8–11]. Several hypotheses suggest that changes in the remineralization length scale within OMZs may promote enhanced carbon retention. For example, reduced zooplankton-driven particle fragmentation in low-oxygen waters has been linked to greater transport of intact particles to depth, thereby enhancing long-term carbon storage [3, 12, 13]. Studies in the eastern tropical North Pacific OMZ observed a decreased attenuation rate of sinking flux, attributed to reduced oxidation of organic matter under hypoxic conditions [14]. Numerous studies have attributed enhanced carbon storage under hypoxic (< 63 *μ *M O_2_) and anoxic (0 *μ *M O_2_) conditions to reduced aerobic mineralization [15–22]. Aerobic transformations, such as aerobic respiration and nitrification, are typically replaced in anoxic conditions by anaerobic pathways, including methanogenesis, denitrification, anammox, and dissimilatory nitrate reduction to ammonium, when oxygen concentrations fall below functional thresholds for respiratory oxidases [23–26]. However, canonical respiratory thresholds indicate that aerobic respiration is not inhibited until oxygen concentrations approach the Pasteur point (*∼ *2.2 *μ *M O_2_) with some indicating thermodynamic favorability for respiration at suboxic conditions (< 1 *μ *M O_2_, or the nitrogenous zone) [26–30]. This discrepancy suggests that oxygen limitation suppresses key oxygen-dependent enzymatic reactions at hypoxic concentrations well above the respiratory threshold, thereby reducing aerobic remineralization of organic matter prior to the limitation of respiration (inhibition of terminal respiratory oxidases). More recent work has reported reduced organic matter degradation within OMZs but has not yet resolved the underlying causes or whether mechanisms differ across gradients from hypoxic to suboxic and anoxic conditions [31]. The observations summarized above point toward increased carbon preservation within OMZs, but lack a clear mechanistic framework explaining how oxygen concentrations directly constrain organic matter transformation in the range of hypoxia.

In the hypoxic barrier hypothesis (HBH) it was proposed that the catabolic degradation of organic matter by non-respiratory oxidases is sensitive to oxygen concentrations in the range of hypoxia and suboxia, impacting the trajectories of ecosystems in oxygen decline [32]. In this scenario, as oxygen concentrations decline to hypoxia and suboxia, they fall below the O_2_  *K_M_* values of oxygen-dependent catabolic enzymes (median value of $\sim \!$ 67 *μ *M) inhibiting their activity, and resulting in the accumulation of oxygen-dependent DOM, defined here as ODDOM. This biochemical threshold may delineate zones of DOM preservation, wherein ODDOM compounds accumulate. In an idealized system where there are no diffusion barriers, net DOM oxidation rates should only fall as systems approach suboxic and anoxic conditions due to metabolic shifts from aerobic to anaerobic activity. Alternatively, if the HBH is correct, then ODDOM compounds will accumulate when oxygen declines into the hypoxic range, even though respiration and other aerobic processes remain active. Because hypoxic waters occupy a much greater volume of the global ocean than anoxic waters and are expanding [32, 33], ODDOM preservation under hypoxic conditions would imply that oxygen-dependent DOM transformations operate over a far broader spatial scale than previously appreciated. This could significantly revise our understanding of ocean carbon storage and the role of microbial metabolism in regulating DOM persistence [34].

We hypothesize that if DOM catabolism is not impacted until oxygen concentrations approach near-anoxic levels and inhibit respiration directly, the canonical assumption is that DOM composition and oxygen consumption should remain unchanged across the hypoxic range. Here, we report results from a 100-day mesocosm incubation experiment designed to simulate a water mass experiencing sustained hypoxia during the summer upwelling season off the Oregon coast [7, 35]. The incubation was conducted with coastal marine communities along with naturally occurring DOM under hypoxic and oxic conditions. Non-targeted metabolomics analysis identified metabolites that accumulated under hypoxic conditions, relative to oxic controls. To compare the chemical signature of these metabolites to predicted ODDOM, metabolites were mapped against a curated database of Kyoto Encyclopedia of Genes and Genomes (KEGG) compounds composed of metabolites that depend on non-respiratory oxidase enzymes for catabolic degradation. To test these predictions, we compared our results with field-derived ODDOM and measured the oxygen dependence of respiration in coastal microbial communities supplied with either ODDOM compounds we identified or common labile DOM (LDOM) compounds that did not rely on non-respiratory oxidases for degradation (Fig. 1A). These experiments suggest that DOM is selectively degraded between hypoxic and oxic conditions, providing mechanistic evidence that low-oxygen inhibition of non-respiratory catabolic oxidases drives ODDOM persistence. These insights are useful for refining biogeochemical models and predicting carbon cycling under expanding hypoxic marine conditions driven by climate change and other anthropogenic influences.

**Figure 1:**
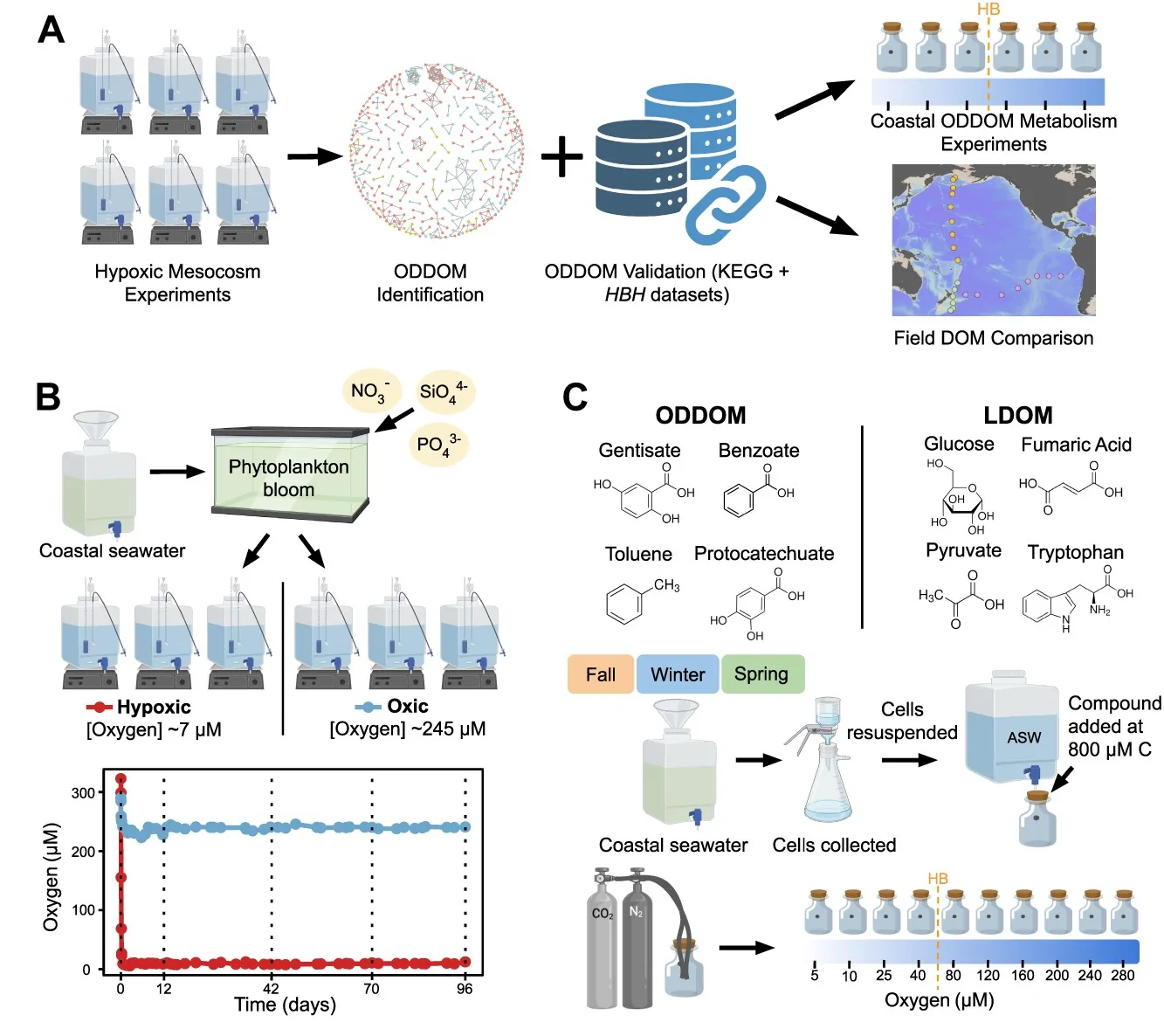
Experimental design of mesocosms and oxygen utilization experiments. (A) Illustration of the study workflow. Hypoxic mesocosm experiments and field datasets were used to identify molecules accumulating under low-oxygen conditions. These molecules were validated using the KEGG database and screened for respiration potential with the HBH database [32]. Identified oxygen-dependent dissolved organic matter (ODDOM) compounds were subsequently evaluated for microbial metabolism in coastal communities under varying oxygen conditions to test the HBH. (B) Experimental setup for mesocosms. Seawater was collected through 20 *μ *m mesh and a phytoplankton bloom was induced in the coastal mixed community. The mixed community was split up into six mesocosm tanks with three receiving low-oxygen treatment (*∼ *7 *μ *M O_2_) and three receiving oxic treatment (*∼ *245 *μ *M O_2_) for 96 days. Metabolomics was sampled five times throughout the experiment (dashed vertical lines: Day 0, 12, 42, 70, and 96). (C) Compounds with ODDOM-like chemical characteristics were identified through analysis of our mesocosm metabolomics dataset, KEGG meta-analysis, and non-respiratory oxidase data from the HBH database [32]. Inhibition of oxygen utilization below the hypoxic barrier was validated using respiration assays on coastal mixed microbial communities exposed to these compounds across a variety of oxygen concentrations.

## Materials and methods

### Mesocosm incubation

Seawater was collected on 27th June 2022 from Yaquina Bay at Hatfield Marine Science Center in Newport, Oregon, USA, during high tide (44$^{\circ }$ 37$^\prime $ N, 124$^{\circ }$02$^\prime $ W, Temp: 12$^{\circ }$C, Time: 1:20 p.m.). Water was filtered through 100 *μ *m nylon mesh into ten 20 L carboys and pumped into a 200 L carboy to homogenize the sampled water (Fig. 1B). Nutrients were added to reach the following concentrations: 441 *μ *M NaNO_3_, 8.1 *μ *M NaH_2_PO_4_H_2_O, 53 *μ *M Na_2_SiO_3_ 9H_2_O, and 100 mL trace element solution (Guillard and Hargraves, 1993). Lights were set to 800 *μ *E/m2/s to simulate Oregon coastal light patterns on a 19-5-h light-dark cycle. Chlorophyll *a* (Chl *a*) concentration was used to monitor the density of the bloom. See Supplemental Methods Section 1.1 for methods of Chl *a* measurement.

After the induced bloom reached its peak after 4 days and Chl *a* started to decline, the water was partitioned into six 20 L carboys (*n* = 3 per treatment, oxic vs hypoxic). The experimental setup was similar to as previously described in [36]. The oxic control treatment consisted of three carboys connected to an aquarium air pump to deliver saturated air filtered through an activated charcoal hydrocarbon trap and a 0.2 *μ *m filter to maintain approximately 245 *μ *M O_2_. For the hypoxic treatment, mass flow controllers were used with mixed gas with 0.05% CO_2_, 2.1% O_2_, and 97.9% N_2_ (Alicat Scientific, Tucson, AZ, USA) and maintained at approximately 7 *μ *M O_2_. Oxygen concentrations were recorded using optode sensors (PreSens, Regensburg, Germany) [37]. Both mesocosm treatments were held at these conditions at 16$^{\circ }$C in the dark for 96 days and sampled regularly for microbial community, nutrients, TOC, metabolites, and oxygen utilization (Supplementary Table S1; Fig. 1B). Mesocosm temperatures were held slightly above in situ conditions to enhance activity and maintain functional niches. One liter of water was collected from each carboy at five time points (Days 0, 12, 42, 70, and 96) in a polypropylene carboy for non-targeted metabolomics. Microbial biomass was collected onto a 0.22 *μ *m pore-size Sterivex filter (polyethersulfone membrane, Millipore, Burlington, MA, USA) using a three-channel peristaltic pump (flow-rate 30 mL/min). 1.5 mL of sucrose lysis buffer was added to the filters before storage at $-80^{\circ }$C. Nucleic acids were extracted using a phenol:chloroform protocol described previously [38, 39]. The V4 region (primers 515F–806R) of the 16S rRNA gene was amplified by PCR and sequenced using individually barcoded universal primers on a MiSeq System (Illumina), following protocols described by the Earth Microbiota Project, at the Center for Quantitative Life Sciences (Oregon State University, Corvallis OR) [40].

### 16S rRNA gene amplicon analyses

Raw forward and reverse sequencing reads were processed in QIIME2 (v2024.10.1) [41]. First, reads were trimmed using cutadapt (v4.9) [42] with default settings. Processed reads were further trimmed, denoised, and chimera removed through DADA2 (v2024.10.0) [43] and assigned taxonomic classification (see Supplemental Methods Section 1.2 for details). Microbial beta-diversity was assessed through Bray-Curtis dissimilarity on non-metric multidimensional scaling [44]. Differences in beta diversity were tested with permutational multivariate analysis of variance (PERMANOVA) using the adonis2 function of vegan (version 2.6-2) in R, after verifying with ANOVA that dispersion between treatments did not differ [45]. Alpha diversity was measured as the exponential Shannon diversity index using vegan in R to represent the effective number of ASVs within each sample.

### Nutrient and TOC measurements

Samples were collected in high-density polyethylene vials for nitrate, nitrite, phosphate, and ammonium measurements. Nutrient samples were sent for analysis at the UCSB Marine Science Institute Analytical Laboratory. Samples were analyzed via flow injection analysis on a Lachat QuikChem 8500 Series 2 by the University of California, Santa Barbara Marine Science Institute Analytical Laboratory (http://analab.msi.ucsb.edu/). High-temperature combustion was used to directly measure TOC content according to details previously described (see Supplemental Methods Section 1.3 for detailed methods) [46].

### Oxygen utilization rates

To determine mesocosm respiration rates, 13 mL samples (ground glass stoppered vials) were collected in triplicate from each carboy twice weekly until Day 24 and then weekly until Day 96. Samples were collected through an integrated sampling port following flow-through equilibration to minimize exposure to atmospheric oxygen. Vials were covered in foil to maintain dark conditions and placed in a 16$^{\circ }$C water bath. Oxygen concentrations were measured using PreSens optode sensors [model: Oxy-4 SMA (G3)] (PreSens, Regensburg, Germany) [37]. The linear slope of oxygen concentrations vs time was used to derive the oxygen utilization rate (Supplementary Table S2). Oxygen rates with linear correlation coefficients below 0.7 were removed from the dataset. One-sided t-tests were used to determine statistical differences between treatments at each timepoint with Bonferroni corrections and a paired t-test between treatments across all timepoints (Supplementary Table S3) [47].

### Non-targeted metabolomics

One liter of 0.22 *μ *m omnipore filtrate (Millipore) was collected at time points 0, 12, 42, 70, and 96 days from each mesocosm. DOM was acidified (pH *∼ * 2) with 12 N HCl and extracted via solid-phase extraction (SPE) with Priority PolLutant (PPL) cartridges (1 g Bond Elut, Agilent) as previously described [48]. DOM was eluted with 2 bed volumes ($\sim\!\! $ 6 mL) of 100% methanol (LC-MS grade) and dried using high purity grade nitrogen and resuspended in 1 mL 80% LC-MS grade methanol. Analysis of PPL-extracted DOM was performed by non-targeted liquid chromatography-tandem mass spectrometry (LC–MS/MS) in data dependent acquisition mode with a Vanquish UHPLC system coupled to a Q-Exactive HF mass spectrometer (see Supplemental Methods Section 2.1 for detailed methods) [49]. LC–MS/MS data were processed via feature-based molecular networking with mzmine and GNPS2, and compound classes were determined from MS/MS spectra using the SIRIUS and CANOPUS workflows (see Supplemental Methods Section 2.2 for details) [50–52].

Molecular abundance was normalized to each sample as percent abundance. Chemical characteristics such as hydrogen to carbon (H/C), oxygen to carbon (O/C), nominal oxidation state of carbon (NOSC), double bond equivalence (DBE), normalized unsaturation (DBE/C), aromaticity index (AI_mod_), molecular lability boundary (MLB: H/C / total number of molecules), and inverse Simpson diversity were calculated based on the SIRIUS molecular formula annotations [48, 53–55]. Weighted values for each molecule were calculated by multiplying the chemical characteristic with the molecular relative intensity. The sums of these weighted values for each molecule within each time point were compared using a paired t-test between treatments. Differences in DOM chemical composition were tested with PERMANOVA using the adonis2 function of vegan (version 2.6-2) in R. Differentially abundant molecules between treatments were determined using a paired t-test (*P* < 0.05) and log2 fold change (> 0.585). Pearson correlation within each treatment and Spearman correlation between treatments were determined for each molecule.

Molecules were sorted into labile DOM (LDOM_M_: abundance decreasing in both treatments), semi-labile to recalcitrant DOM (SLRcDOM_M_: abundance increasing in both treatments), and ODDOM_M_ (abundance increasing/remaining the same in hypoxic conditions and decreasing in oxic conditions) (Supplementary Fig. S1A). All DOM derived groups from the mesocosm datasets are annotated with a subscript M. Detailed methods of DOM classification can be found in Supplemental Methods Section 2.3. Visualization of these criteria can be found in the Supplementary Fig. S1A. SIRIUS annotations for molecules within each DOM class were used for statistical comparison of chemical characteristics using the Wilcoxon rank sum test in R [56]. For DOM composition analysis, molecular abundances were aggregated based on CANOPUS superclass annotations within each DOM class (LDOM_M_, SLRcDOM_M_, or ODDOM_M_).

All enzymatic reactions involving oxygen in KEGG (database release v110) were compiled [57]. Reactants with O_2_ were filtered, and respiratory quinones and other respiratory oxidase reactions were removed, resulting in a database of catabolic oxidase reactants (Supplementary Table S4). The reactants of non-respiratory oxidases with known O_2_  *K_M_* values were obtained from a previously curated database [32]. Compounds were converted into PubChem IDs using the tool MetaboAnalyst 5.0 [58]. PubChem IDs were converted into international chemical identifiers in PubChem [59] and the chemical formulas were extracted. All statistical comparisons of chemical characteristics between datasets were made in R using the Wilcoxon rank sum test (Fig. 2).

**Figure 2:**
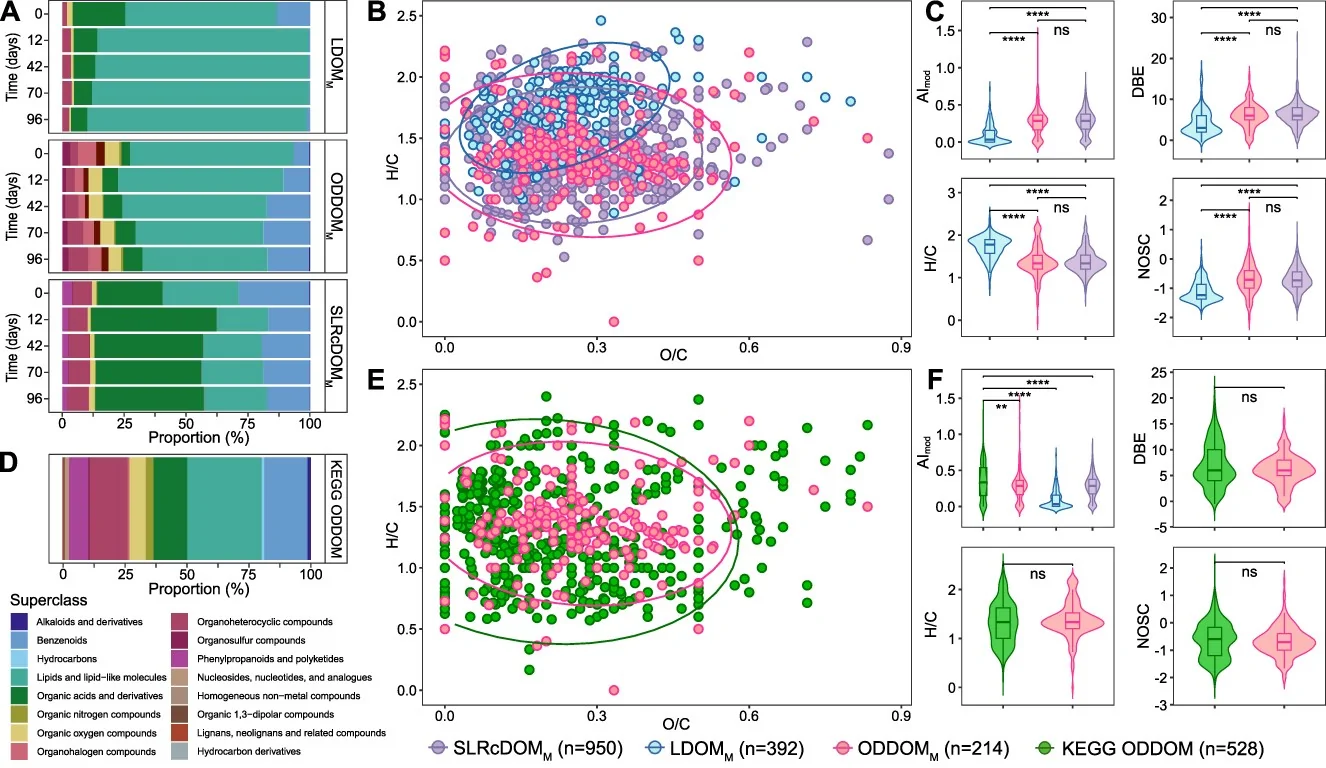
Identifying ODDOM molecules in mesocosm experiments. (A) Superclass composition of DOM classified as LDOM_M_, ODDOM_M_, or SLRcDOM_M_ within the mesocosm hypoxic treatment through time. (B) Van Krevelen plot of all molecules classified as LDOM_M_, ODDOM_M_, and SLRcDOM_M_. (C) Statistical comparison of chemical characteristics of each DOM type (LDOM_M_: Labile DOM, ODDOM_M_: Oxygen-dependent DOM, and SLRcDOM_M_: Semi-labile to recalcitrant DOM). Chemical characteristics: H/C, NOSC, DBE, AI_mod_. All comparisons were conducted using the Kruskal-Wallis test with *P* value significance indicated. (D) Superclass composition of DOM classified as ODDOM from the KEGG database. (E) Van Krevelen plot of experimentally identified ODDOM (EXP ODDOM_M_) and ODDOM identified in KEGG (KEGG ODDOM). (F) Statistical comparison of experimental DOM datasets (ODDOM_M_, LDOM_M_, and SLRcDOM_M_) to ODDOM identified in KEGG. Statistical tests between two datasets were conducted using Wilcoxon rank-sum test and statistical test comparing all four datasets was conducted using Kruskal-Wallis test. *P* value significance indicated as ns: *P*  *≥ * 0.05, ^*^: *P* < 0.05, ^*^^*^: *P*  *≤ * 0.01, ^*^^*^^*^: *P*  *≤ * 0.001, ^*^^*^^*^^*^: *P*  *≤ * 0.0001.

### FT-ICR-MS meta-analysis

The molecular signatures of metabolites identified by LC-MS/MS in our experiments were further compared with 220 samples collected from the Pacific Ocean (7 stations and 18 samples under hypoxia) for Fourier transform ion cyclotron resonance mass spectrometry (FT-ICR-MS) (Fig. 3) during three cruises aboard the R.V. Sonne: SO245 (95–165$^{\circ }$ W, 20–40$^{\circ }$ S), SO248 (30$^{\circ }$–50$^{\circ }$ N), and SO254 (30–50$^{\circ }$ S) [60]. A summary of the dataset can be found here https://doi.pangaea.de/10.1594/PANGAEA.962747. All samples were extracted using the SPE method as previously described [48]. Due to low coverage sampling below 2000 m across stations, samples collected from depths below 2000 m were excluded from further analysis. For FT-ICR-MS data, molecular relative intensities were normalized by formula peaks, and ratios of H/C and O/C were calculated for each molecule [61]. DBE, DBE/C, NOSC, and AI_mod_ of the molecular formulas were calculated from the number of atoms of each element as previously described [48, 53, 54]. Molecular formulas with relative intensities that increased with depth were categorized as recalcitrant DOM candidates (RcDOM_E_: Spearman *∣ **r**∣ * > 0.3, *P* < 0.05). Molecular formulas that were dominant in the surface samples (< 10 m, > 70% non-zero formula intensity) but vanishing at depth (> 1000 m, < 30% non-zero formula intensity) were categorized as labile DOM (LDOM_E_). Samples with similar depth profiles (between 500 and 1000 m) and varying oxygen content (22.98 *μ *M to 230.63 *μ *M O_2_) were used for identifying ODDOM_E_ (Fig. 2B). Molecular formulas whose relative intensities showed a significant negative correlation with oxygen concentrations were categorized as ODDOM_E_ (Spearman *r*  *< -0.3*, *P* < 0.05). All DOM derived groups from the environmental datasets are annotated with a subscript E.

**Figure 3:**
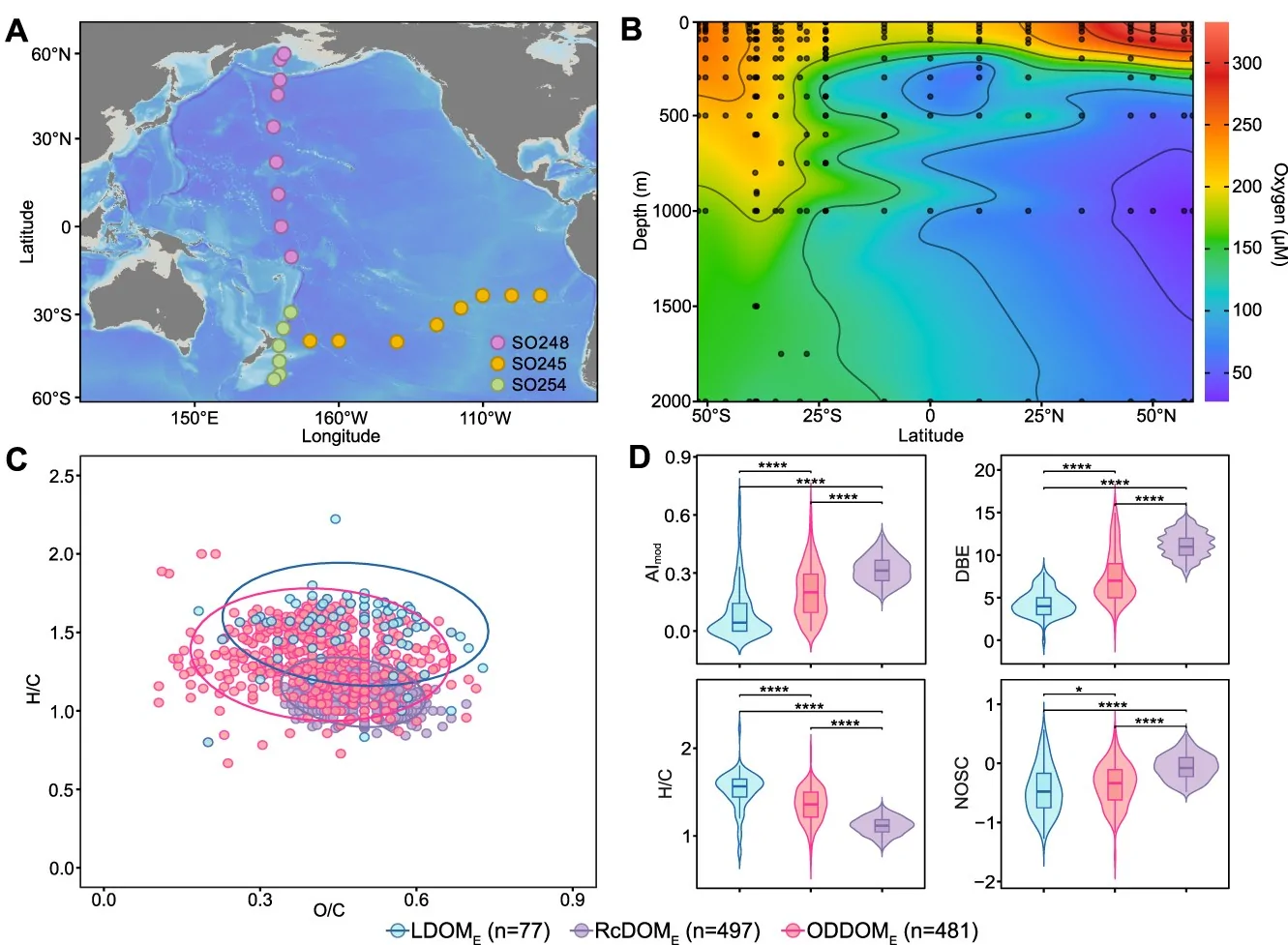
Classification of environmental ODDOM in FT-ICR-MS data using oxic and hypoxic ocean ecosystems. (A) Transect lines for three cruises (SO245, SO248, and SO254) aboard the R.V. Sonne that collected FT-ICR-MS data utilized in this analysis [60]. (B) Oxygen concentrations from each collection point with respect to depth and latitude. (C) Van Krevelen plot of all molecules classified as LDOM_E_ (labile DOM), ODDOM_E_ (oxygen-dependent DOM), and RcDOM_E_ (recalcitrant DOM candidates) based on their relative intensity in relation to their depth distribution or the oxygen concentrations in the environment (see methods for details). (D) Statistical comparison of chemical characteristics of each DOM type identified from the environment (LDOM_E_, ODDOM_E_, and RcDOM_E_). Chemical characteristics: H/C, NOSC, DBE, AI_mod_. All comparisons were conducted using the Kruskal-Wallis test with *P* value significance indicated as ns: *P*  *≥ * 0.05, ^*^: *P* < 0.05, ^*^^*^: *P*  *≤ * 0.01, ^*^^*^^*^: *P*  *≤ * 0.001, ^*^^*^^*^^*^: *P*  *≤ * 0.0001.

### Respiration rate experiments

Two groups of compounds—selected based on whether their metabolism was predicted to be inhibited under low-oxygen conditions or not—were added in pure form to natural marine microbial communities in artificial seawater (ASW) to test the oxygen dependence of community respiration rates (Fig. 1B) [62]. ODDOM compounds that were selected for testing met these criteria: they shared functional groups/structures with ODDOM_M_ (LC-MS/MS features identified as ODDOM in the mesocosm experiments; Supplementary Fig. S2); they were included among the ODDOM compounds predicted from the KEGG database (Supplementary Fig. S3); and oxidases involved in their catabolism were previously reported in the biochemical literature to have high K_*M*_ values [32]. LDOM compounds were selected for having catabolic pathways in KEGG that are independent of oxygen with the exception of respiratory oxidases. Naturally occurring marine microbial communities were collected from South Beach in Newport, Oregon, USA (44$^{\circ }$36$^\prime $ N 124$^{\circ }$04$^\prime $ W) across three different seasons (see Supplementary Table S5 for details). Seawater was filtered, and cells were collected and resuspended in ASW before respiration experiments began (see Supplemental Methods Section 3.1 for details). Respiration experiments were conducted using the automated biological oxygen demand (AutoBOD) system from the Van Mooy lab at Woods Hole Oceanographic Institution, which enabled high-temporal resolution oxygen consumption measurements to estimate whole-community O_2_  *K_M_* values. The system includes a 12-bottle (168 mL volume each) rotating carousel for 48–96 h of continuous incubation and oxygen monitoring. Ten bottles were bubbled with a mixture of CO_2_ (0.051%) and N_2_ (99.95%) to the desired oxygen level ranging from 5 to 280 *μ *M O_2_ amended with tested compounds (*∼ *800 *μ *M carbon) and starved cells ($\sim\kern-6.5pt 2\times $10^5^  *- 2× *10^6^ mL^-1^) alongside two oxic controls with ASW amended with cells or compounds only. Cell abundance was measured using a Guava easyCyte HT Flow Cytometer (Millipore, Burlington, MA), and pH was measured using an OrionVersaStar Pro pH meter (Thermo Fisher Scientific, Waltham, MA). Details of each experiment’s carbon source, oxygen levels, cell counts, and pH can be found in Supplementary Tables S5 and S6.

Community respiration apparent O_2_ affinity constants (whole-community O_2_ K_*M*_) were determined by measuring rates of oxygen consumption in samples that spanned a range of starting oxygen concentrations (see Supplemental Methods Section 3.2 for details). Bottles that reached near 0 *μ *M and had average starting concentrations closest to 40 *μ *M O_2_ were used for kinetic model fitting (Supplementary Tables S7 and S8). The inflection point at which the linear rate of oxygen utilization became exponential decay was visually selected as the starting timepoint for each respiration assay for model fitting (20 *μ *M < O_2_ < 40 *μ *M) due to the varied delay of cell activity depending on the community and compound tested. The final time point was selected as the point in which oxygen reached less than 3 *μ *M O_2_. Oxygen concentration vs time was fitted to two models; the Single-phase Exponential Model, indicating monophasic kinetics and the Sigmoidal Gompertz Decay Model, indicating biphasic kinetics (see Supplemental Methods Section 3.2).

## Results and discussion

### Mesocosm dissolved organic matter composition

DOM composition was studied over 100 days in mesocosms after an induced phytoplankton bloom, in triplicate hypoxic and oxic treatments. The initial TOC concentration in the bloom water was 387.3 *± * 5.4 *μ *M. TOC rapidly declined to an average of 198.5 *± * 16.1 *μ *M by Day 12 in the hypoxic treatment and 190.5 *± * 21.9 *μ *M in the oxic treatment, and then decreased gradually to an average of 143.0 *± * 5.5 *μ *M by Day 96 in the hypoxic treatment and 139.1 *± * 2.9 *μ *M in the oxic treatment (Supplementary Fig. S4A). Overall, TOC concentrations were significantly higher in the hypoxic treatment compared to oxic treatment from Days 12 to 96 (Supplementary Fig. S4A; *P* =0.043; Supplementary Table S9).

We hypothesized that non-respiratory catabolic oxidase inhibition by hypoxia would cause the accumulation of ODDOM compounds. A total of 8,567 metabolites were identified by LC–MS/MS and compared across treatments using weighted averages of chemical characteristics. NOSC, DBE, and AI_mod_ were higher under hypoxic conditions but did not show significant differences (*P* = 0.11, 0.15, and 0.053, respectively; Supplementary Fig. S4A; Supplementary Table S10). Overall DOM composition did not differ significantly between treatments based on measured chemical characteristics (PERMANOVA, treatment: *P* = 0.34); however, by the end of the incubation, hypoxia increased TOC storage by 2.6% (*P* = 0.043) (Supplementary Fig. S4A). Principal component analysis (PCA) revealed that incubation time was the biggest factor in determining DOM composition (PERMANOVA, time: *P* < 0.001), but within each time point, the treatments separated along a similar axis. NOSC and AI_mod_ were the strongest predictors of DOM divergence between the treatments (Supplementary Fig. S4B). These findings indicate that hypoxia did not have a strong influence on the overall DOM composition, but may act on a distinct set of compounds that drive the differences observed between treatments. These compounds had a higher oxidation state, greater unsaturation, and higher aromaticity, features that are typically associated with less bioavailable DOM [63–65]. Similar trends were observed in the Yangtze River Estuary, where hypoxia was associated with increased DOM unsaturation and aromaticity, suggesting that low-oxygen conditions facilitate the preservation of polyphenolic terrestrial carbon [9]. In the seasonally hypoxic Bedford Basin an accumulation of recalcitrant-like DOM was associated with declining oxygen levels, including elevated aromaticity at O_2_ concentrations below ca. 100 *μ *M [66]. The inhibition of non-respiratory oxidase activity whose O_2_  *K_M_* values exceed the oxygen conditions thereby constraining the catabolism of these relative recalcitrant compounds has been proposed as a mechanism for the accumulation of transformed DOM with similar chemical structures [32].

Metabolites were further categorized into different DOM categories based on their temporal abundance patterns: LDOM_M_ (decreased in both treatments; *n* = 551), ODDOM_M_ (decreased only under oxic conditions; *n* = 291), and SLRcDOM_M_ (increased in both treatments; *n* = 1,295) (Supplementary Fig. S1A). A conceptual model illustrating categorical DOM preservation within the mesocosms is presented in the Supplementary Fig. S1B. Metabolite superclasses revealed the relative chemical structure of each DOM category (Fig. 2A). LDOM_M_ was dominated by lipid-like compounds, whereas ODDOM_M_ and SLRcDOM_M_ were enriched in ring-containing compounds (i.e. benzenoids and organoheterocyclic compounds) (Fig. 2A). ODDOM_M_ and SLRcDOM_M_ shared similar positions in van Krevelen plots, whereas LDOM_M_ was shifted upward to higher H/C ratios (Fig. 2B). Comparisons of the chemical characteristics of molecules identified by LC-MS/MS revealed no significant difference between the ODDOM_M_ and SLRcDOM_M_ sets (*P* >0.05), whereas the LDOM_M_ molecules on average had significantly higher H/C but lower AI_mod_, DBE, and NOSC, in line with the more reduced and labile characteristics expected for this set of molecules (all *P* <0.05) (Fig. 2C) [65].

The relative intensity of oxygen-containing compounds was highest in ODDOM_M_ and the proportion of benzenoid and heterocyclic compounds increased over time relative to LDOM_M_. Counterintuitively, many substrates for oxidases are stable, oxygenated compounds that are degraded via non-respiratory oxidase-catalyzed reactions (Supplementary Fig. S3). ODDOM compounds predicted by KEGG pathways (those with non-respiratory oxidase enzymatic reactions) were not significantly different from experimentally identified ODDOM_M_ molecules in H/C, NOSC, and DBE (*P* >0.05) (Fig. 2F). Likewise, KEGG-defined and experimentally identified ODDOM were similar when overlayed on van Krevelen plots, and the percentage contribution of the superclasses benzenoids, oxygen-containing, and heterocyclic were similar, suggesting experimentally identified ODDOM_M_ molecules were chemically similar to ODDOM compounds predicted in silico from KEGG (Fig. 2E). Although the AI_mod_ differed significantly between the two groups (*P*  *≤ *0.01), the experimentally identified ODDOM_M_ molecules showed the highest overall similarity to KEGG-defined ODDOM when compared to all other experimentally defined DOM groups (Fig. 2F). This suggests that ODDOM exhibits a high degree of aromaticity, with oxygen-limited conditions below the hypoxic barrier (*∼ *67 *μ *M O_2_) promoting a directional increase in DOM aromatic content. Elevated aromaticity reflects enhanced molecular stability and is characteristic of DOM fractions that likely require specialized catabolic oxidase activity for microbial degradation [65, 67, 68]. ODDOM_M_ molecules identified in the mesocosm experiments were not significantly different chemically (AI_mod_, DBE, H/C, and NOSC) to the set of molecules we defined as SLRcDOM_M_ based on their persistence independent of oxygen concentrations (Fig. 2C). Recalcitrant DOM is proposed to consist of a group of molecules that have high environmental persistence either because they are intrinsically resistant to enzymatic degradation because of thermodynamic barriers [69], or because they are too diluted to provide net positive benefits to cells when metabolized [70, 71]. If these ODDOM compounds are resistant to degradation under hypoxic conditions due to inhibition of catabolic oxidases, this would provide direct evidence that intrinsic molecular characteristics of the DOM, through specific substrate–enzyme interactions, contribute to their resistance to microbial breakdown [70–72]. However, if oxic conditions are restored, we would predict that these ODDOM compounds become fully degradable, suggesting that the persistence of certain organic matter pools is contingent on environmental conditions.

### Validation of ODDOM in mixed community respiration

Two sets of model compounds were selected for respiration assays in natural communities based on criteria that predicted whether their metabolism would be inhibited by hypoxia or not (see methods for details): LDOM (a monosaccharide, an aromatic amino acid, and two organic acids) and ODDOM (4 oxygenated benzenoids; Supplementary Fig. S3). We hypothesized that if catabolism of these ODDOM compounds depends on non-respiratory oxidases with O_2_  *K_M_* values in the typical range for such enzymes (*∼ * 67 *μ *M O_2_) then oxygen consumption rates should decrease below this value. This is a theoretical expectation based on enzyme kinetics and it is unknown at the level of community function whether cells have mechanisms that overcome non-respiratory oxidase inhibition by hypoxia, such as increasing the expression of the affected enzymes or shifting to alternative pathways [26, 73, 74]. The O_2_ K_*M*_’s estimated from Michaelis-Menten curves and Lineweaver-Burk plots for each compound (Fig. 4) were significantly lower for LDOM (average *K_M_* = 2.11 *μ *M O_2_) than for ODDOM (average *K_M_* = 68.67 *μ *M O_2_) (Fig. 4B; *P* = 0.0015). The empirically determined community-average *K_M_* for the tested ODDOM compounds (68.67 *μ *M) is nearly identical to the mean *K_M_* of non-respiratory oxidases across the Tree of Life [32] (67 *μ *M O_2_) (Fig. 4B; Supplementary Fig. S5).

**Figure 4:**
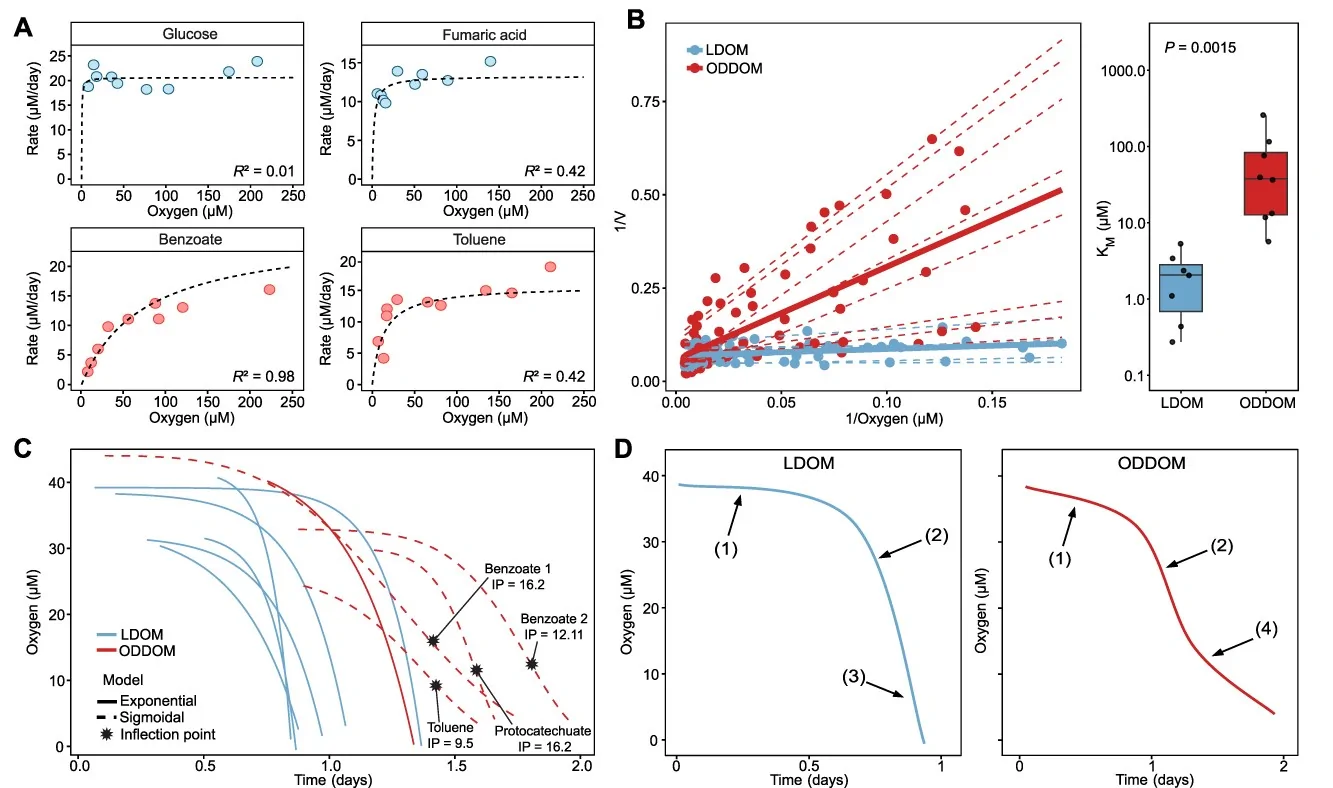
ODDOM validation in seawater respiration experiments. (A) Oxygen utilization Michaelis-Menten curves for LDOM (blue) and ODDOM (red) compounds with *R*^2^ reported (all additional compound Michaelis-Menten curves can be found in Supplementary Fig. S5; all KEGG pathways used to determine if compounds are LDOM or ODDOM can be found in Supplementary Fig. S3). (B) Lineweaver-Burk plot of all compounds tested with ODDOM compounds in red and LDOM compounds in blue. Boxplot with statistical comparison (Wilcoxon signed-rank test) of calculated O_2_ K_*M*_ values derived from the lineweaver-Burk plot between LDOM and ODDOM compounds. LDOM average *K_M_* = 2.11 *μ *M O_2_, ODDOM average *K_M_* = 68.67 *μ *M O_2_. (C) Best fit model to either exponential decay or sigmoidal decay for each compound’s oxygen utilization. Compound type (LDOM or ODDOM) is indicated by line color and best fit model is indicated by line type. Details on the selection of the best fit model can be found in Supplementary Table S11. (D) Conceptual model illustrating monophasic and biphasic oxygen kinetics of microbial oxidases: microbial respiration supported by LDOM exhibits monophasic kinetics, whereas metabolism of ODDOM compounds requiring non-respiratory catabolic oxidases displays biphasic kinetics: (1) constant rate of oxygen utilization before exponential decay caused by growth and/or acclimation; (2) exponential culture growth results in a rapid increase in the rate of oxygen utilization, (3) respiration increases exponentially until O_2_ reaches nanomolar levels (< 350 nM O_2_) with little to no evidence of inhibition; (4) sigmoidal curve with second inflection, attributed to non-respiratory oxidase inhibition. We attribute the second inflection in each case, all of which occurred below the proposed hypoxic barrier (*∼ *67 *μ *M O_2_), to non-respiratory oxidase inhibition.

In the respiration assays that were initiated just below hypoxia (*∼ * 40 *μ *M), oxygen consumption data were fitted to exponential and sigmoidal decay models to reveal the oxygen utilization kinetics associated with each compound’s metabolism. Depending on best fit, each compound was designated monophasic or biphasic kinetics (Fig. 4D). All but one ODDOM respiration assay was a better fit to a sigmoidal curve, indicating biphasic kinetics; as oxygen levels dropped below a threshold, oxygen consumption slowed (Fig. 4C; see Supplementary Table S11 for model fit results). These results suggest that as oxygen concentrations decline below the *K_M_* values of active non-respiratory oxidases, cells are unable to compensate, and the lessened activity of the catabolic pathway causes diminished electron flow to respiration. In contrast, all LDOM compounds were best described by exponential decay, reflecting oxygen consumption without a threshold effect (Fig. 4C; Supplementary Table S11). In these cases, even as oxygen concentrations declined to and below the hypoxic barrier, oxygen consumption was sustained, suggesting that the metabolism of these compounds was unimpeded by low oxygen until anoxia was reached. These results demonstrate that microbial respiration of a portion of DOM, here termed ODDOM, is reduced when oxygen levels fall below the hypoxic barrier, whereas respiration of LDOM can continue until near-anoxic conditions are reached [26, 32, 74, 75]. Although whole-community respiration assays inherently encompass substantial biological complexity, making it difficult to disentangle microbial adaptive responses and community turnover effects on oxygen consumption, the consistent patterns observed across multiple microbial communities strengthen our confidence in the robustness and generality of this conclusion. Alternative explanations for the declines in oxygen consumption during ODDOM metabolism under hypoxia, such as microbial cells switching to anaerobic catabolic pathways to degrade these compounds, are plausible [76]. Alternative pathways to degrade aromatic compounds have been identified, such as the benzoyl-CoA pathway, but require anoxic conditions [77, 78]. ODDOM substrates were selected because no oxygen-independent degradation pathways for these compounds are currently annotated in the KEGG database. If such metabolism occurred in these experiments, it could involve previously uncharacterized enzymes or alternative metabolic routes operating under hypoxic conditions capable of aromatic ring degradation.

### Identification of ODDOM in public environmental data

A meta-analysis of FT-ICR-MS datasets was conducted to evaluate the presence and characteristics of ODDOM in natural environments (Fig. 3). Features were grouped based on sampling depth and oxygen concentrations based on expectations of each category (see methods section FT-ICR-MS Meta-analysis). Van Krevelen plots of these groups revealed patterns similar to those observed in the mesocosm experiments, with ODDOM_E_ overlapping with both RcDOM_E_ and LDOM_E_ (Fig. 3C). Across all molecular characteristics (AI_mod_, DBE, NOSC, and H/C) ODDOM_E_ occupied an intermediate position between LDOM_E_ and RcDOM_E_, suggesting it may represent an intermediate DOM pool with conditional lability (Fig. 3D) [70]. Prior studies have documented the accumulation of recalcitrant DOM-like characteristics within DOM pools of coastal hypoxic zones [9, 66]. During tropical cyclones in the Eastern Tropical North Pacific (ETNP), researchers found molecules associated with recalcitrant DOM characteristics, including terpenes and lactones, to be ubiquitous across the oxic to anoxic water column [79]. We found that ODDOM shared molecular characteristics with SLRcDOM, but no abiotic or biochemical pathway has been identified that would lead from ODDOM to recalcitrant DOM, and the relationship between these carbon pools remains a point of conjecture. One interpretation is that ODDOM resists degradation within coastal OMZs, leading to an apparent expansion of the recalcitrant DOM pool. In recent work, DOM from suboxic water in Devil’s Hole, Bermuda, incubated with surface microbial communities stimulated the growth of taxa capable of degrading recalcitrant-like DOM (e.g. SAR202) and showed increased catabolic oxidase activity [11]. These results suggest that ODDOM preserved under low-oxygen conditions can remain bioavailable once oxic conditions are restored. However, it remains unclear whether prolonged exposure to hypoxic conditions, beyond seasonal hypoxia or suboxia, alters the recalcitrant characteristics of ODDOM, warranting further investigation. Vast metabolic niches exist within OMZs and redox water columns that may provide greater insight into the metabolic transformation of ODDOM [80–82]. For example, sulfurization of DOM under hypoxia may further stabilize compounds identified here as ODDOM [82, 83].

### Community composition and response to hypoxia

Throughout the mesocosm experiments, both time and oxygen conditions significantly shaped changes in the composition of the heterotrophic microbial community, with increasing divergence between treatments over time (PERMANOVA, treatment: *P* < 0.004, time: *P* < 0.001) (Fig. 5B). Alpha diversity of the heterotrophic community increased significantly over the first two weeks of the experiment but subsequently declined in both treatments (Fig. 5D). The parallel decline between treatments suggests a bottle effect that promoted similar succession and turnover trajectories of the community. The heterotrophic microbial community at the start of the mesocosm experiments was dominated by members of *Rhodobacterales* and *Flavobacterales* (26.11 *± * 12.67% and 29.13 *± * 13.72% relative abundance at T0 respectively) (Fig. 5A). These groups are commonly associated with phytoplankton blooms and have distinctive arrays of carbohydrate transporters [84]. The relative abundance of photosynthetic lineages decreased throughout the dark incubation, consistent with expectations (Supplementary Fig. S6). SAR86 emerged as the most abundant taxonomic group under hypoxic conditions at Day 42, whereas the oxic treatment was dominated by *Pelagibacterales* (SAR11) (Fig. 5A). This pattern is consistent with observations reported in similar mesocosm experiments [36].

**Figure 5:**
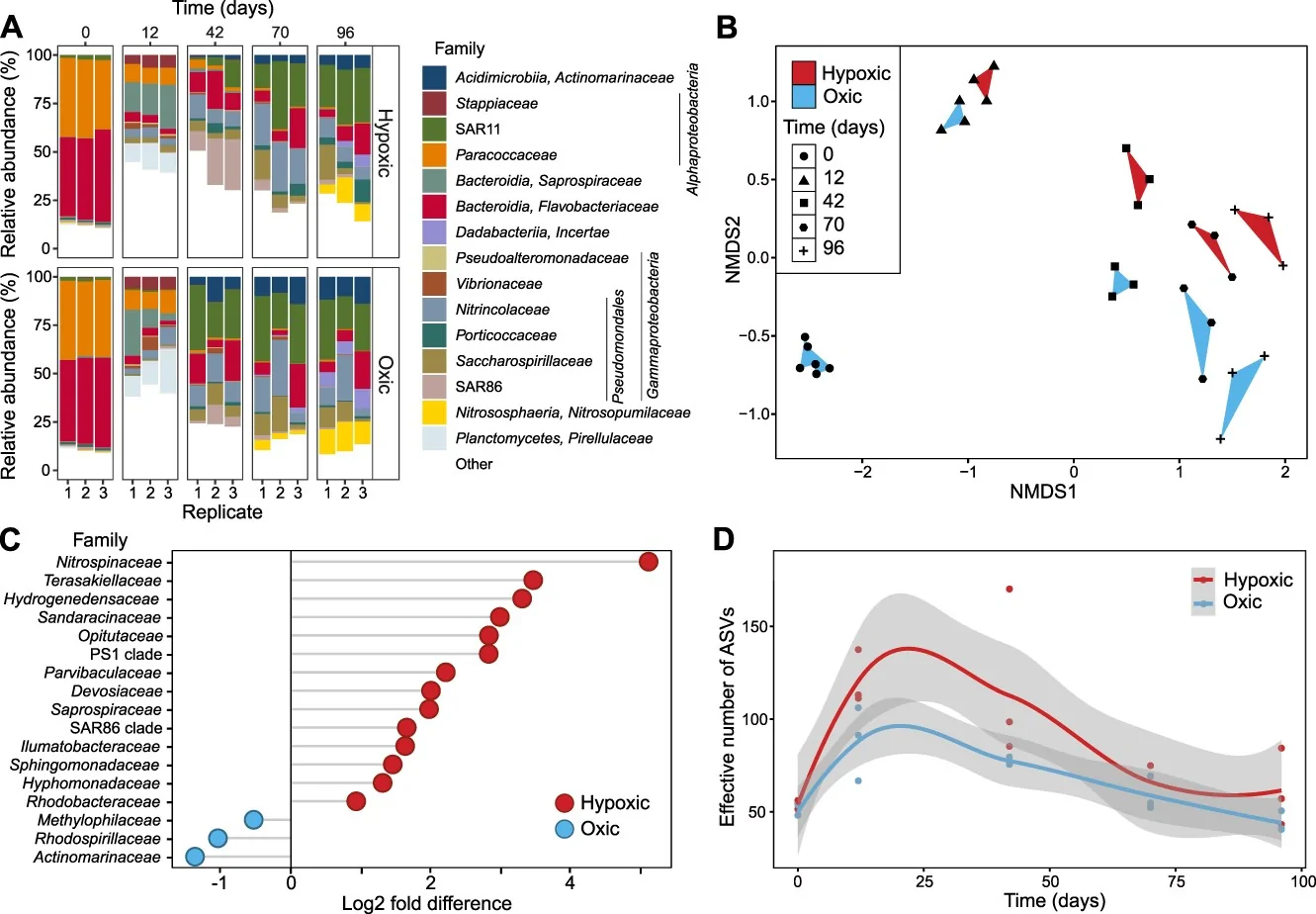
Microbial community composition and structure in mesocosm experiments. (A) Microbial community composition within the mesocosm experiments over time under each treatment. ASVs were aggregated at the family or class level as specified in the SILVA database. (B) NMDS plot of mesocosm community structure through time clustered by treatment. (C) Differential abundance of taxa clustered to family level across treatments from Day 42 to Day 96. The taxa shown differed significantly (*P* < 0.05, log2 fold change > 0.585) between hypoxic and oxic treatments. Positive values indicate taxa that were more abundant in the hypoxic treatment. (D) Exponential Shannon diversity index to represent effective number of ASVs within each treatment through time.

Taxa that were found to have significantly higher relative abundance in either treatment can be found in Supplementary Table S12. Some common, abundant marine taxa, for example *Actinomarinaceae*, *Rhodospirillaceae*, and *Methylophilaceae*, were significantly less abundant in the hypoxic treatment; however, the abundance of other microbial families increased in the hypoxic treatment (Fig. 5C). Previous work reported similar patterns and observed that they sometimes manifested at the level of individual amplicon sequence variants (ASVs) but in other cases appeared to apply broadly at higher taxonomic scales [36]. One interpretation of these patterns is that cells may have evolved diverse adaptations to hypoxic conditions, with many taxa being hypoxia tolerant but others either hypoxia intolerant or to varying degrees microaerophilic [85]. One of these taxa was SAR86 subclade D2472, a highly abundant organism in the surface ocean that has been observed with increased abundance under hypoxia [36] (Supplementary Fig. S7). Adaptations to hypoxia that obviate catabolic oxidase enzyme inhibition have not been described. We hypothesize that such adaptations might exist and play a role in determining microbial growth in hypoxic environments. Future research should link these responsive ASVs to genomic information to identify metabolic traits and dependencies associated with different oxygen conditions.

Around Day 72 of the experiment, aerobic nitrifiers were enriched under hypoxic conditions (Fig. 6C). This was evident by the significant transformation of nitrite to nitrate by Day 96 and a large increase in oxygen utilization in comparison to the oxic treatment from Days 60 to 96 (Fig. 6C). Corresponding to the spike in nitrate, the ASV displaying the highest differential abundance between hypoxic and oxic conditions was *Nitrospinaceae*, with 1.43 *± * 1.56% relative abundance in hypoxic vs 0.08 *± * 0.05% in oxic carboys. *Nitrospinaceae* are known members of the microbial community in OMZs, oxidizing nitrite and actively fixing inorganic carbon in the mesopelagic and within OMZs [79, 86, 87]. Nitrite oxidation genes have been found to be upregulated under nearly anoxic conditions in the ETNP with increased nitrification in suboxic conditions [88]. Collectively, these findings support previous observations that nitrification can proceed efficiently under low-oxygen conditions, facilitated by terminal respiratory oxidases that enable continued oxygen consumption [89]. This dynamic in principle might create a feedback loop that intensifies the proposed accumulation of the ODDOM pool in systems entering hypoxia and aligns with observations of nitrogen cycling and elevated nitrifier abundance across the oxic–suboxic transition zones of oxygen minimum systems [66, 90].

**Figure 6:**
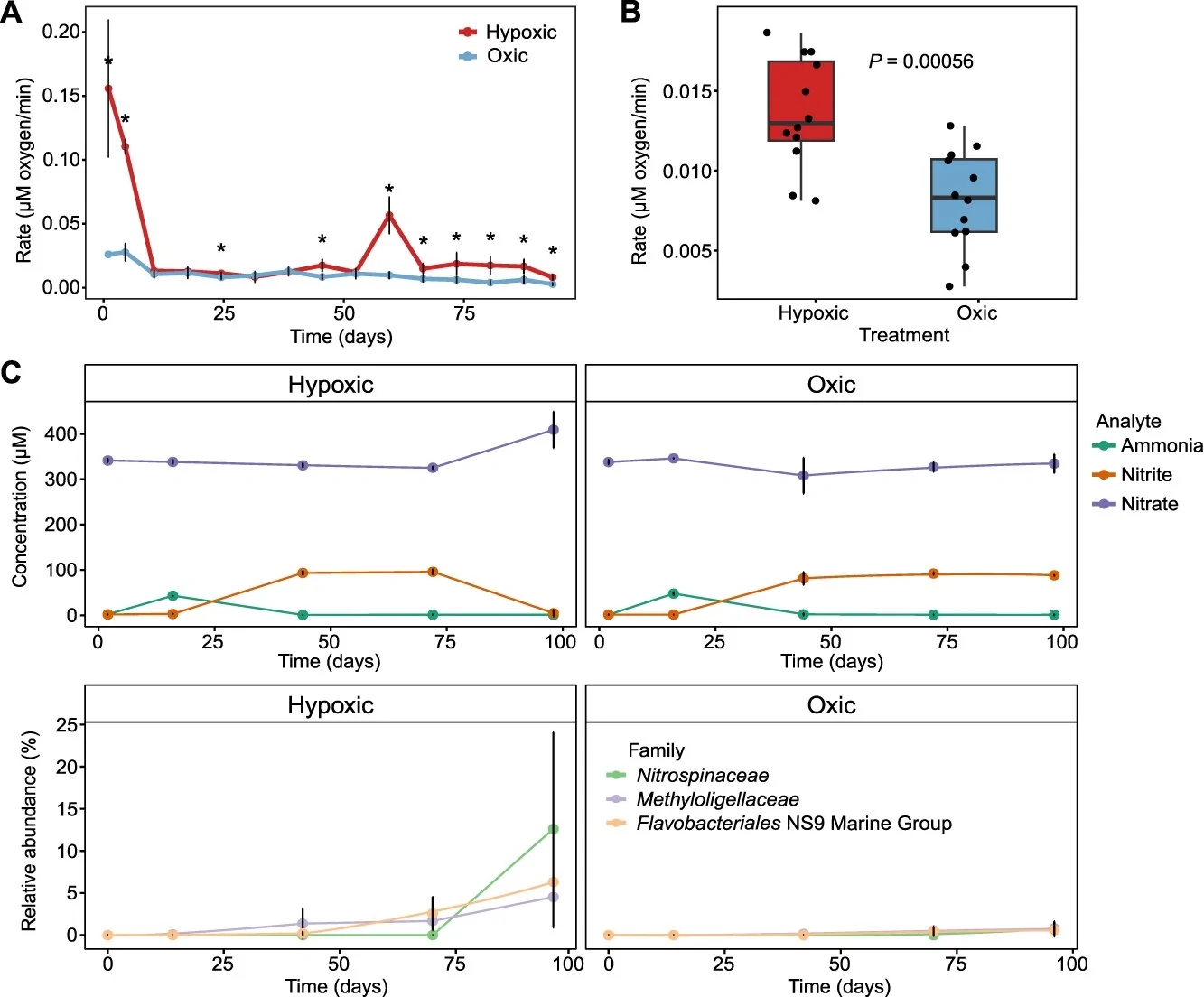
Nitrite oxidation increases under hypoxia. (A) Oxygen utilization rates within each mesocosm treatment through time. Timepoints in which the rate of oxygen utilization was significantly different (*P* < 0.05) are marked with asterisks. (B) Paired t-test of oxygen utilization rates between treatments with rates from outlier timepoints 1, 4.5, and 59.5 removed. (C) Nitrite, nitrate, and ammonia concentrations within the mesocosm through time and the relative percent abundance of taxa that were significantly more abundant in hypoxic conditions at timepoint 96.

Nitrification was postulated to explain a portion of the decoupling between oxygen consumption and carbon oxidation reported in previous studies, since nitrification can support substantial oxygen utilization under favorable conditions [91, 92]. Our results further indicate that nitrogen cycling plays a critical role in shaping community respiration rates and should be explicitly considered when estimating carbon export using respiration quotients [91, 93]. This process may create a feedback that both sustains low-oxygen conditions and promotes the accumulation of ODDOM, thereby enhancing dissolved carbon storage within OMZs and reducing carbon export efficiency in hypoxic waters.

### Implications for marine carbon dioxide removal

Our results suggest that hypoxia inhibits the catabolism of DOM compounds that require non-respiratory oxidases, thereby contributing to the retention of TOC in OMZs during periods of hypoxia (Fig. 7). Although previous studies have emphasized the role of hypoxic upwelling systems in enhancing carbon storage through the transport of particulate organic carbon to depth [94, 95], our findings highlight a complementary mechanism: the suppression of oxygen-dependent catabolic processes that would otherwise degrade DOM. Because many non-respiratory oxidases appear to be required to degrade a large class of compounds (ODDOM), their inhibition under hypoxia may slow the turnover of DOM, allowing carbon to accumulate in the water column. Together, these results provide a mechanistic link between oxygen availability and organic matter preservation, offering a potential explanation for the observed buildup of TOC in hypoxic environments. As hypoxic conditions (< 63 *μ *M O_2_) become more widespread with ongoing climate change, the accumulation of ODDOM is likely to be enhanced. Earth system model projections consistently show that the global ocean experiences continuing declines in oxygen levels over the twenty-first century as a result of climate change, expanding the hypoxic area of marine ecosystems [96]. Thus, increased ocean deoxygenation could serve as a partial buffer to rising atmospheric CO_2_ levels and may help account for the observed annual increase in DOM inputs to the global ocean [2, 7]. The effect of deoxygenation on carbon storage likely depends on the initial DOM composition: waters rich in LDOM may continue respiration to near-anoxic levels if electron flow to terminal respiratory oxidases is sustained. Within the OMZ near Peru and Chile, cells have been shown to shift gene expression to lower O_2_  *K_M_* respiratory oxidases in response to decreasing oxygen, which likely explains the continued consumption of oxygen to near completely anoxic conditions [26, 74, 97]. In contrast, systems dominated by aromatic ODDOM may experience inhibited respiration when oxygen drops below the hypoxic threshold, ultimately contributing to the preservation of carbon in these OMZs. This study identified ODDOM using respiration assays with pure compounds, phytoplankton-derived DOM, and mesopelagic Pacific water (500 to 1000 m); however, these compounds likely represent only a subset of a broader and chemically diverse ODDOM pool whose contribution to total DOM may vary with source and microbial community (e.g. terrestrial or deep-ocean).

**Figure 7:**
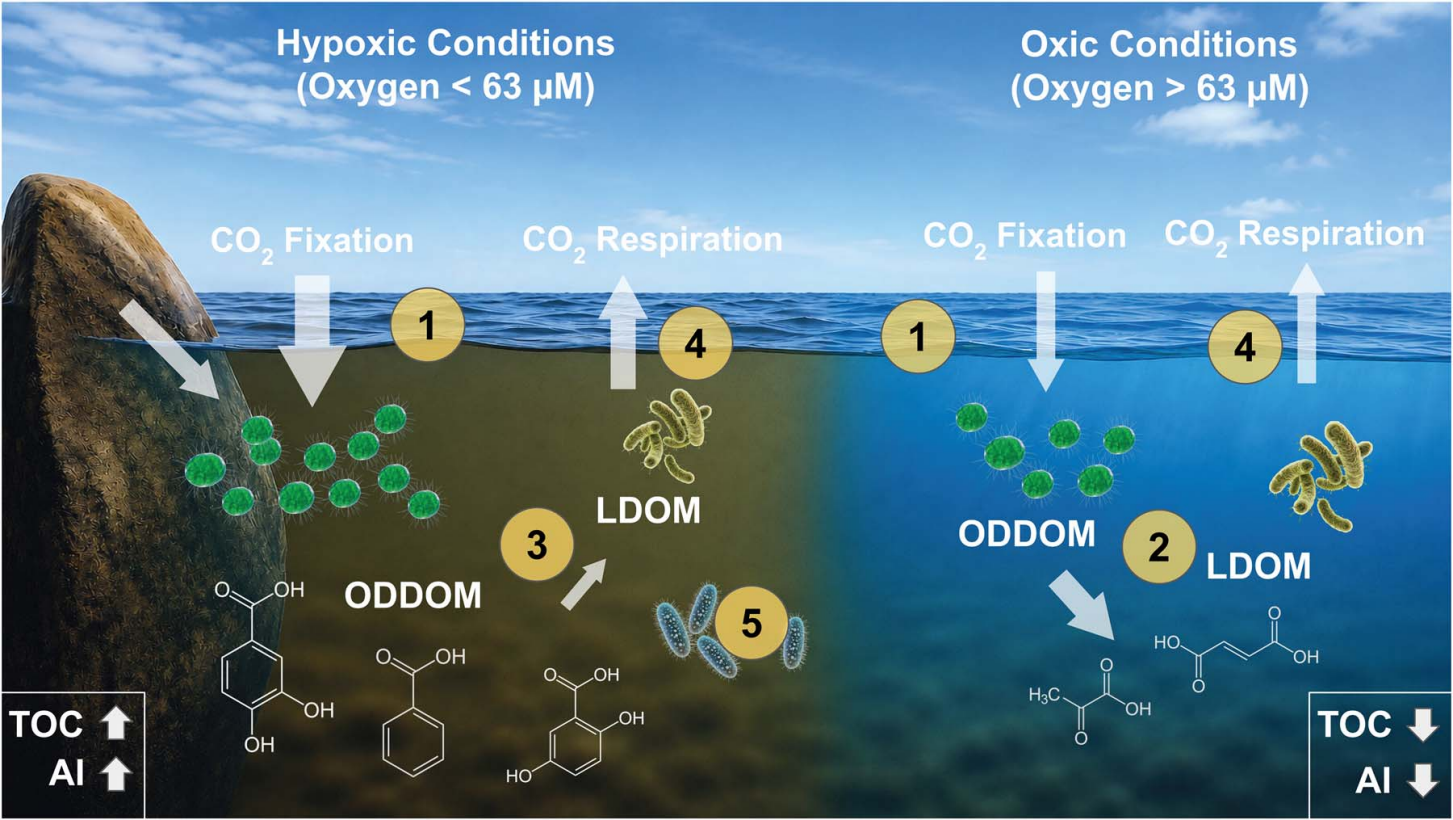
Conceptual model illustrating the limitation of ODDOM catabolism. This conceptual model represents the expected DOM outcomes for water masses that share similar environmental conditions but differ in oxygen levels. TOC and AI were found to be elevated under hypoxic conditions. Chemical structures under hypoxic conditions display representative ODDOM compounds (Supplementary Fig. S2). Chemical structures under oxic conditions display representative LDOM compounds. The thickness of the arrows represents conceptual flux magnitude. (1) Phytoplankton blooms and/or terrestrial organic matter runoff provide a highly diverse DOM pool with increased aromaticity. (2) Under oxic conditions, ODDOM is degraded into labile compounds that heterotrophic bacteria use for cellular respiration, resulting in lower overall TOC and a greater proportion of fixed carbon being converted to CO_2_. (3) Under hypoxic conditions, non-respiratory oxidases are inhibited by low oxygen, leading to an accumulation of ODDOM, overall TOC, and aromaticity of the DOM pool. (4) Cellular respiration occurs using labile organic carbon in both conditions with a larger relative pool of LDOM under oxic conditions due to the degradation of ODDOM. The elevated net primary production associated with hypoxic water masses is expected to result in greater carbon fluxes (indicated by arrow thickness). (5) Under hypoxic conditions, aerobic nitrifying bacteria continue to utilize oxygen, further maintaining low-oxygen conditions.

We describe experimental evidence for a hypothesized class of DOM compounds, referred to as ODDOM, that increases in relative intensity in ecosystems when oxygen declines to hypoxia. The community-average *K_M_* we observed for respiration of model ODDOM compounds (68.67 *μ *M O_2_) closely matches the average *K_M_* of non-respiratory oxidases across the Tree of Life (67 *μ *M O_2_), highlighting a widespread biochemical constraint that emerges near the conventional boundary of hypoxia (63 *μ *M O_2_). Future research should extend this framework to test how oxygen availability and DOM composition shape microbial metabolic responses and adaptations to hypoxia and their consequences for carbon cycling in the ocean. Additionally, future studies should compare DOM composition between environmental samples and experimental mesocosms with matched DOM source material and oxygen conditions to further validate the persistence of ODDOM. The evidence we present for differential impacts of hypoxia on microbial community structure and catabolic oxidase activity suggests that cells might have undescribed metabolic adaptations to oxygen concentrations in this range [76, 90].

## Supplementary Material

Supplementary_material_wrag220

## Data Availability

Amplicon sequence datasets from this study have been deposited in the NCBI SRA database under the BioProject identifier PRJNA1363168. Metabolomic datasets from this study have been deposited in MassIVE under accession MSV000090678. All nutrient, oxygen, and carbon measurements can be found in the Supplementary Tables. All code used for downstream analyses can be found on GitHub at https://github.com/bndaniel/Oxygen-limited-Metabolism-HBH.git.
