# Supplementary material for: Oxygen-dependent metabolism of dissolved organic matter in hypoxic marine environments
Source: ISME J. 2026 Aug 26;20(1):wrag220. doi: 10.1093/ismejo/wrag220 (PMC13630563; doi:10.1093/ismejo/wrag220)
Supplement: Supplementary file 1 [file supplementary_material_wrag220.zip › Supplemental_figures_wrag220.pdf]

Figure S1

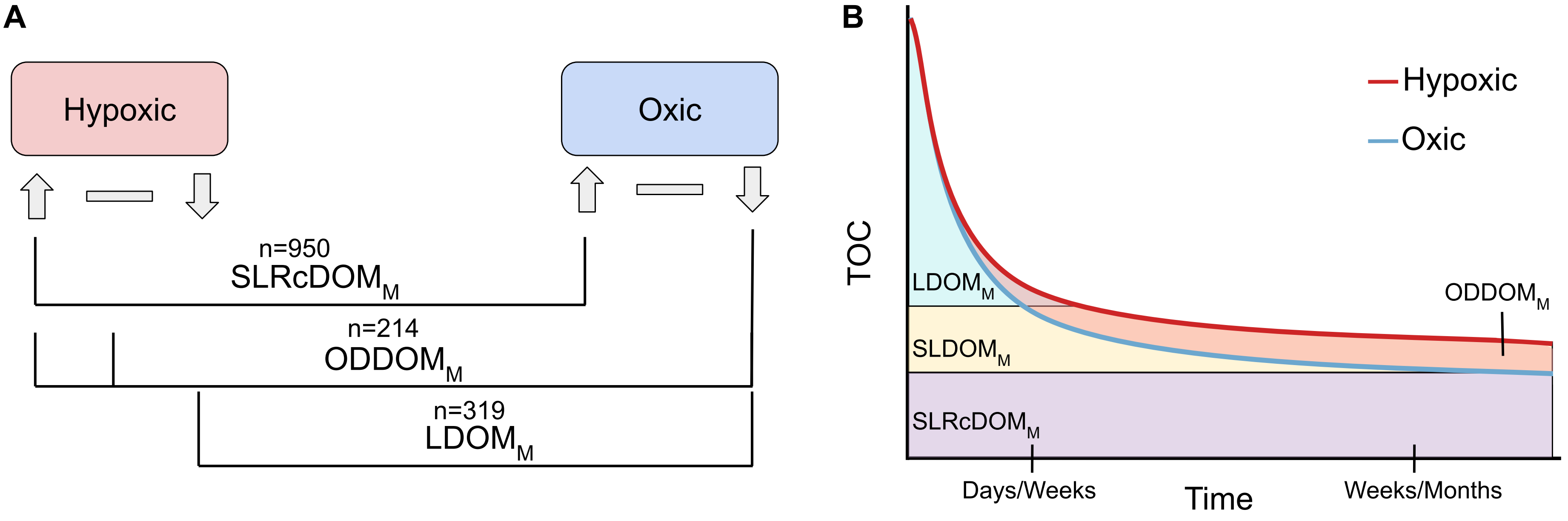

A
Hypoxic
Oxic
n=950
SLRcDOM$_M$
n=214
ODDOM$_M$
n=319
LDOM$_M$
B
Hypoxic
Oxic
TOC
LDOM$_M$
SLDOM$_M$
SLRcDOM$_M$
ODDOM$_M$
Days/Weeks
Time
Weeks/Months

Figure S2

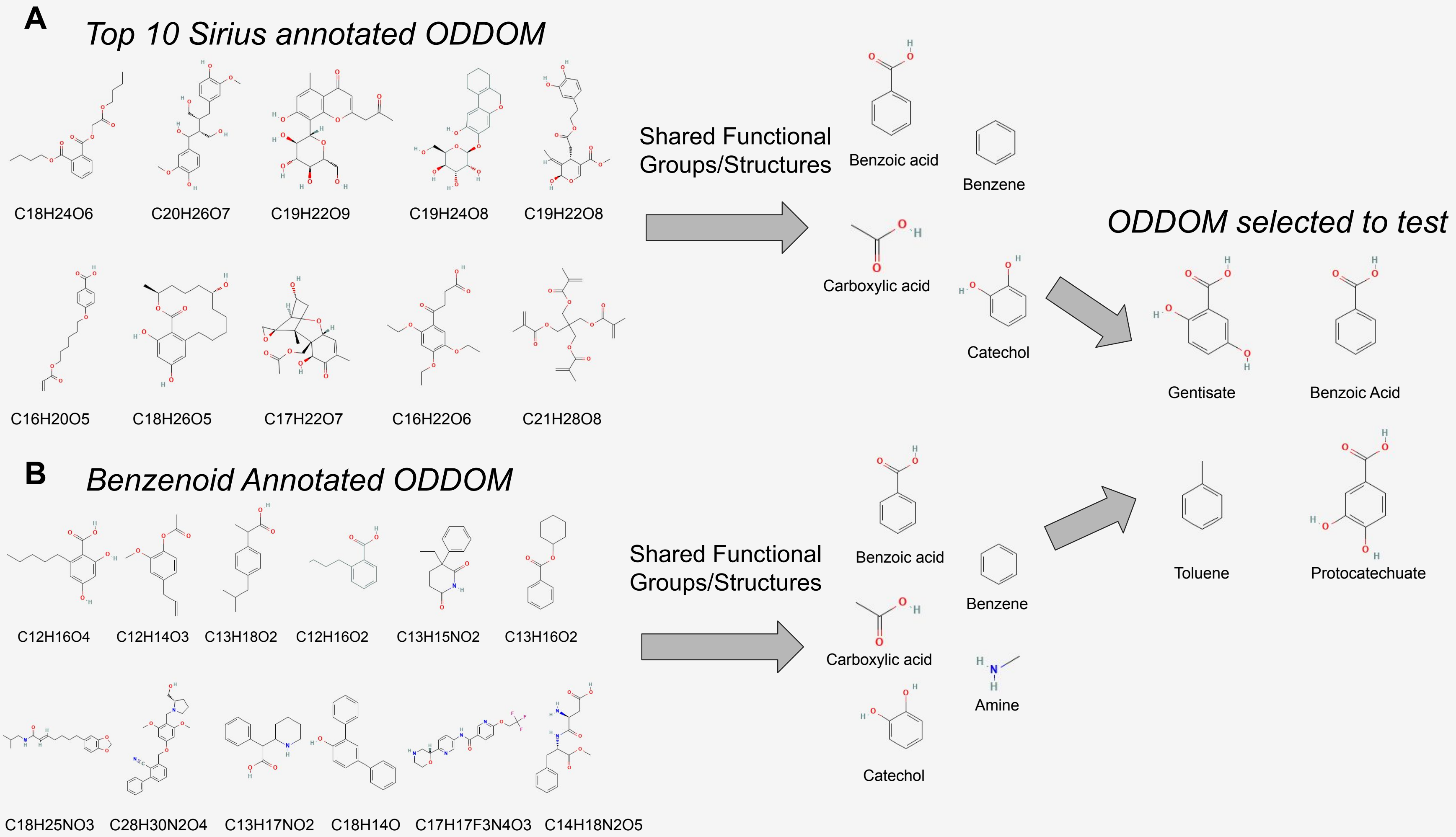
A
Top 10 Sirius annotated ODDOM
C18H24O6
C20H26O7
C19H22O9
C19H24O8
C19H22O8
C16H20O5
C18H26O5
C17H22O7
C16H22O6
C21H28O8
Shared Functional Groups/Structures
Benzoic acid
Benzene
Carboxylic acid
Catechol
ODDOM selected to test
Gentisate
Benzoic Acid
Toluene
Protocatechuate
B
Benzenoid Annotated ODDOM
C12H16O4
C12H14O3
C13H18O2
C12H16O2
C13H15NO2
C13H16O2
C18H25NO3
C28H30N2O4
C13H17NO2
C18H14O
C17H17F3N4O3
C14H18N2O5
Shared Functional Groups/Structures
Benzoic acid
Benzene
Carboxylic acid
Amine
Catechol

Figure S3

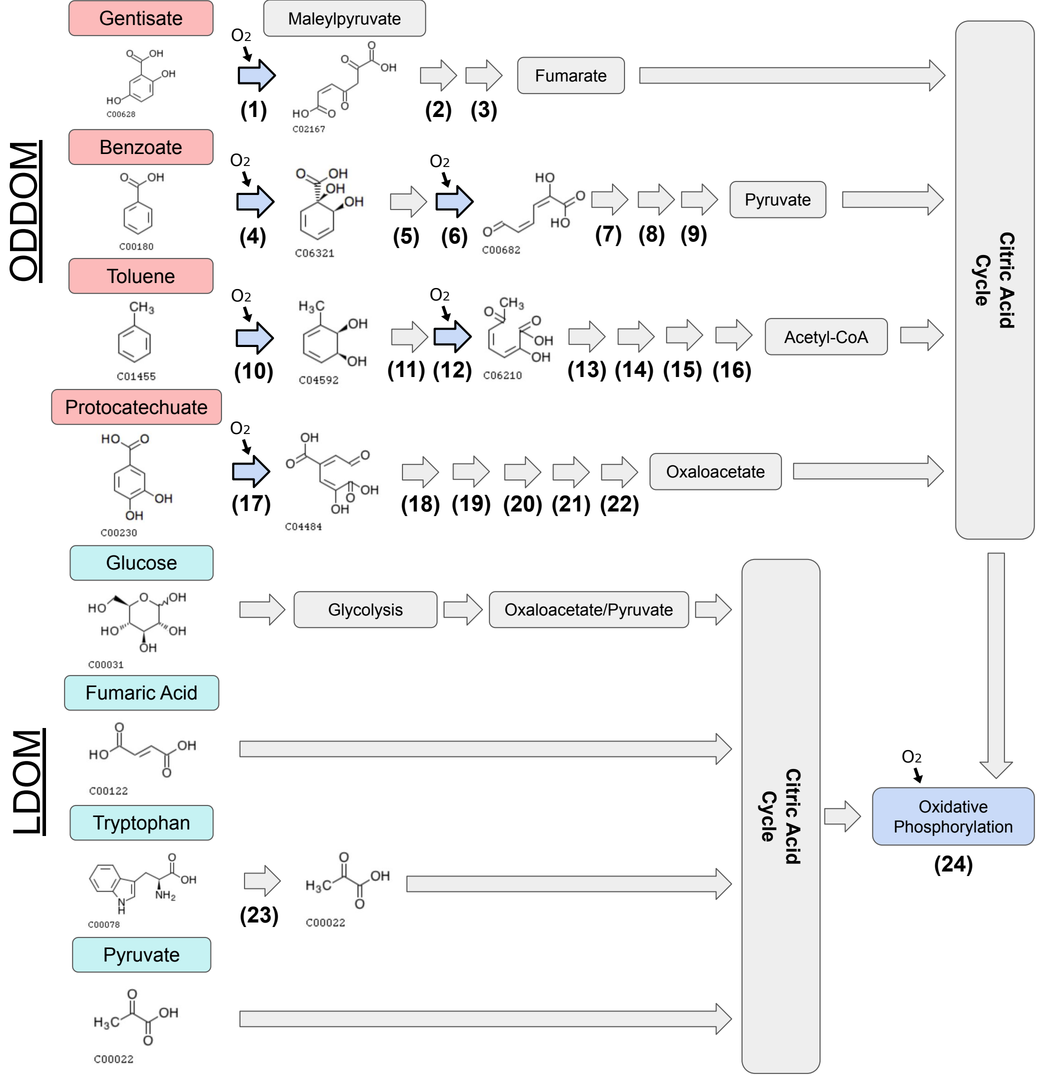

ODDOM
Gentisate
C00628
O2
(1)
Maleylpyruvate
C02167
(2) (3)
Fumarate
Benzoate
C00180
(4)
C06321
(5) (6)
C00682
(7) (8) (9)
Pyruvate
Toluene
C01455
(10)
C04592
(11) (12)
C06210
(13) (14) (15) (16)
Acetyl-CoA
Protocatechuate
C00230
(17)
C04484
(18) (19) (20) (21) (22)
Oxaloacetate
Citric Acid Cycle
LDOM
Glucose
C00031
Glycolysis
Oxaloacetate/Pyruvate
Fumaric Acid
C00122
Tryptophan
C00078
(23)
C00022
Pyruvate
C00022
Citric Acid Cycle
Oxidative Phosphorylation
(24)

Figure S4

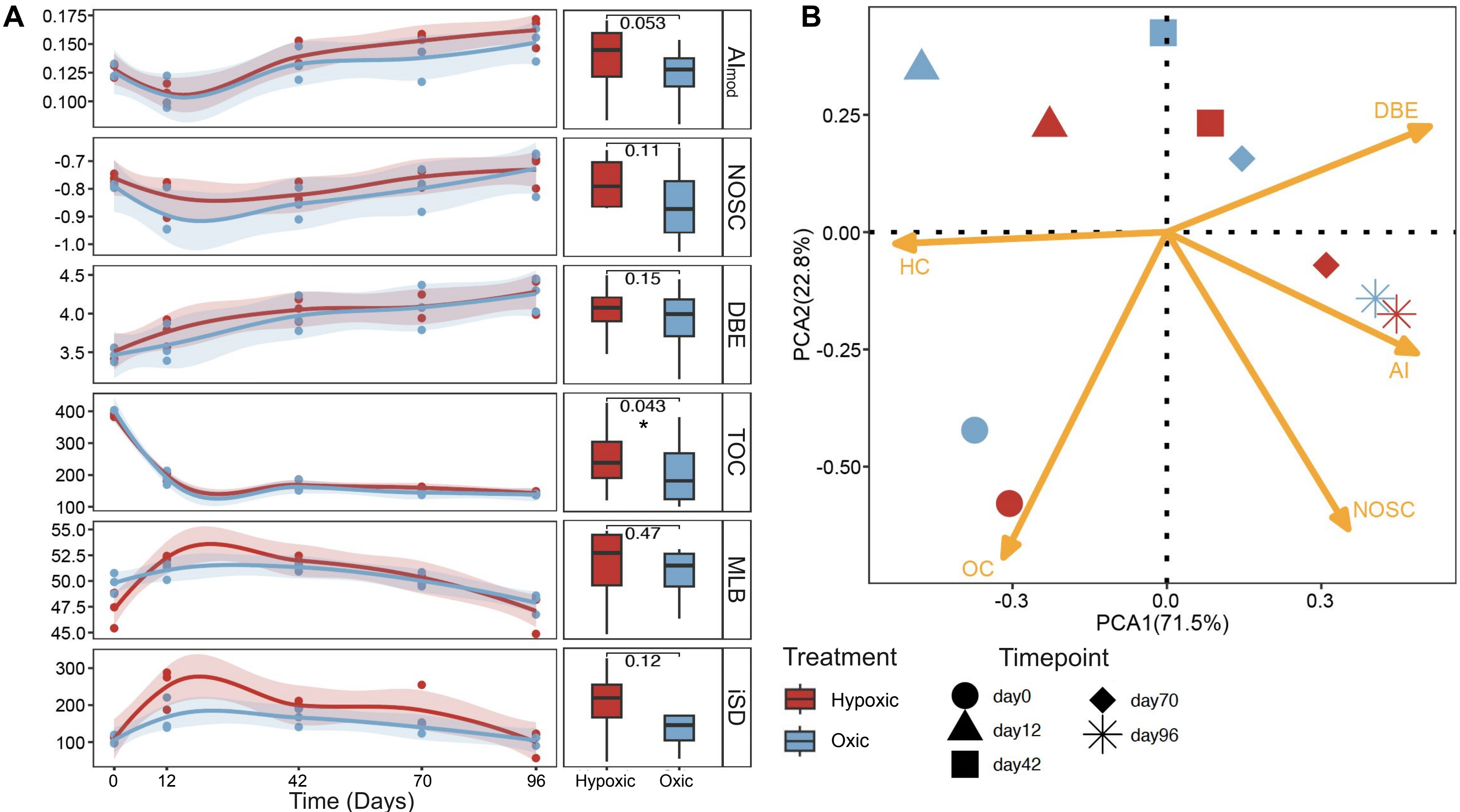

A
0.175
0.150
0.125
0.100
0.053
AI_{mod}
-0.7
-0.8
-0.9
-1.0
0.11
NOSC
4.5
4.0
3.5
0.15
DBE
400
300
200
100
0.043
*
TOC
55.0
52.5
50.0
47.5
45.0
0.47
MLB
300
200
100
0.12
iSD
0
12
42
70
96
Time (Days)
Hypoxic
Oxic
B
0.25
0.00
-0.25
-0.50
PCA2(22.8%)
-0.3
0.0
0.3
PCA1(71.5%)
DBE
HC
AI
NOSC
OC
Treatment
Hypoxic
Oxic
Timepoint
day0
day12
day42
day70
day96

Figure S5

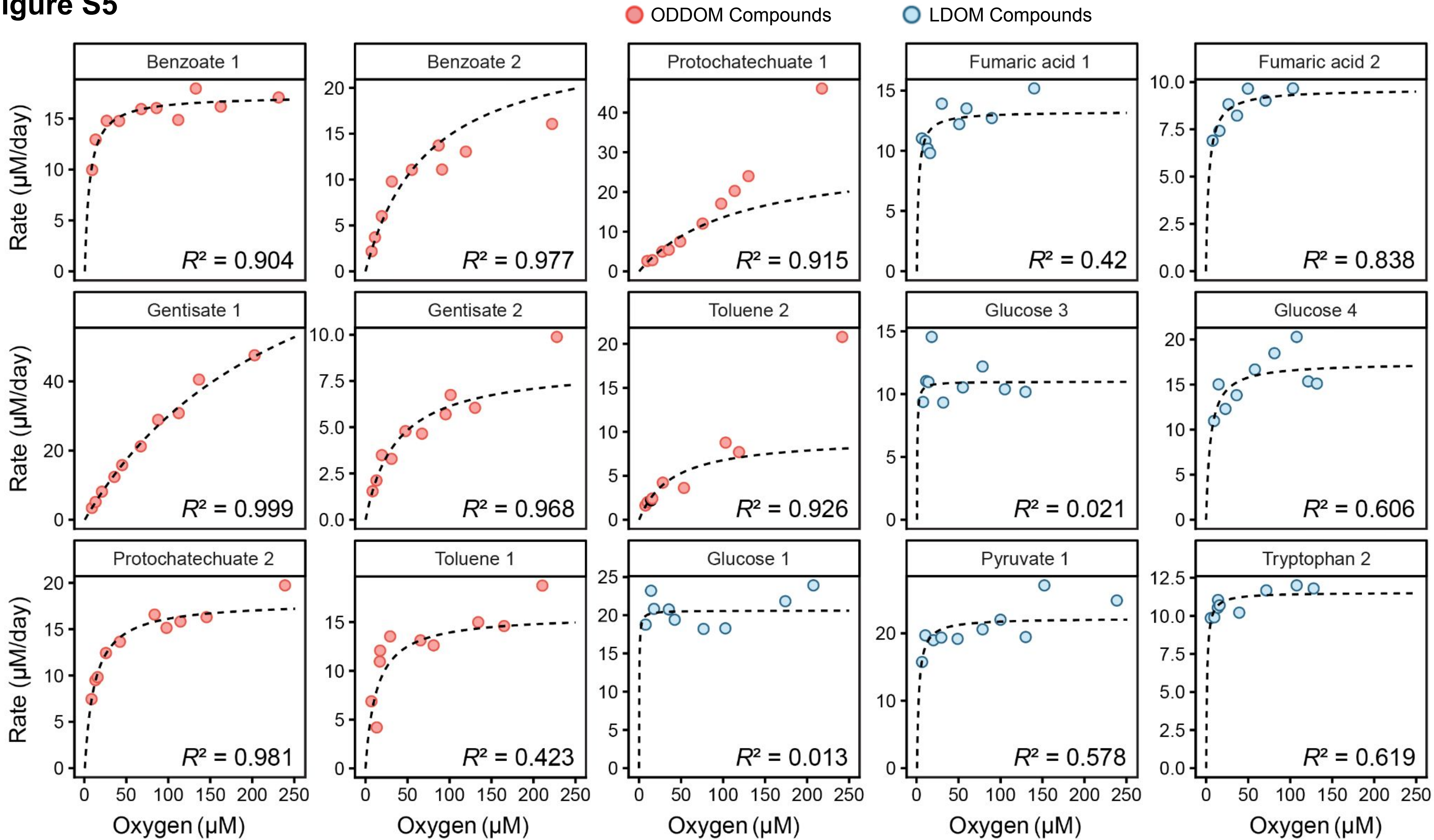

ODDOM Compounds
LDOM Compounds
Benzoate 1
Benzoate 2
Protochatechuate 1
Fumaric acid 1
Fumaric acid 2
Gentisate 1
Gentisate 2
Toluene 2
Glucose 3
Glucose 4
Protochatechuate 2
Toluene 1
Glucose 1
Pyruvate 1
Tryptophan 2
R² = 0.904
R² = 0.977
R² = 0.915
R² = 0.42
R² = 0.838
R² = 0.999
R² = 0.968
R² = 0.926
R² = 0.021
R² = 0.606
R² = 0.981
R² = 0.423
R² = 0.013
R² = 0.578
R² = 0.619
Rate (µM/day)
Oxygen (µM)

Figure S6

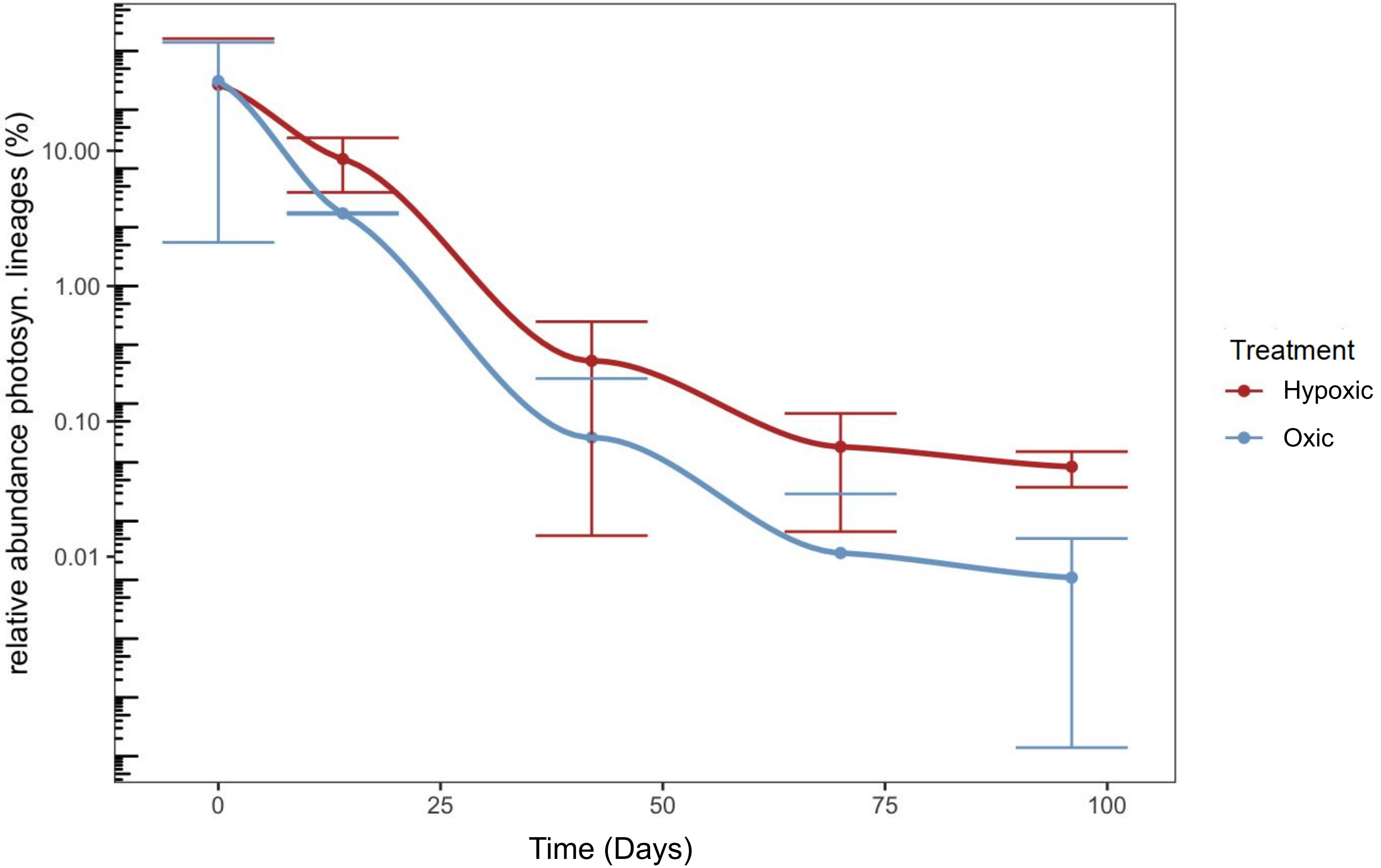

relative abundance photosyn. lineages (%)
10.00
1.00
0.10
0.01
0
25
50
75
100
Time (Days)
Treatment
Hypoxic
Oxic

Figure S7

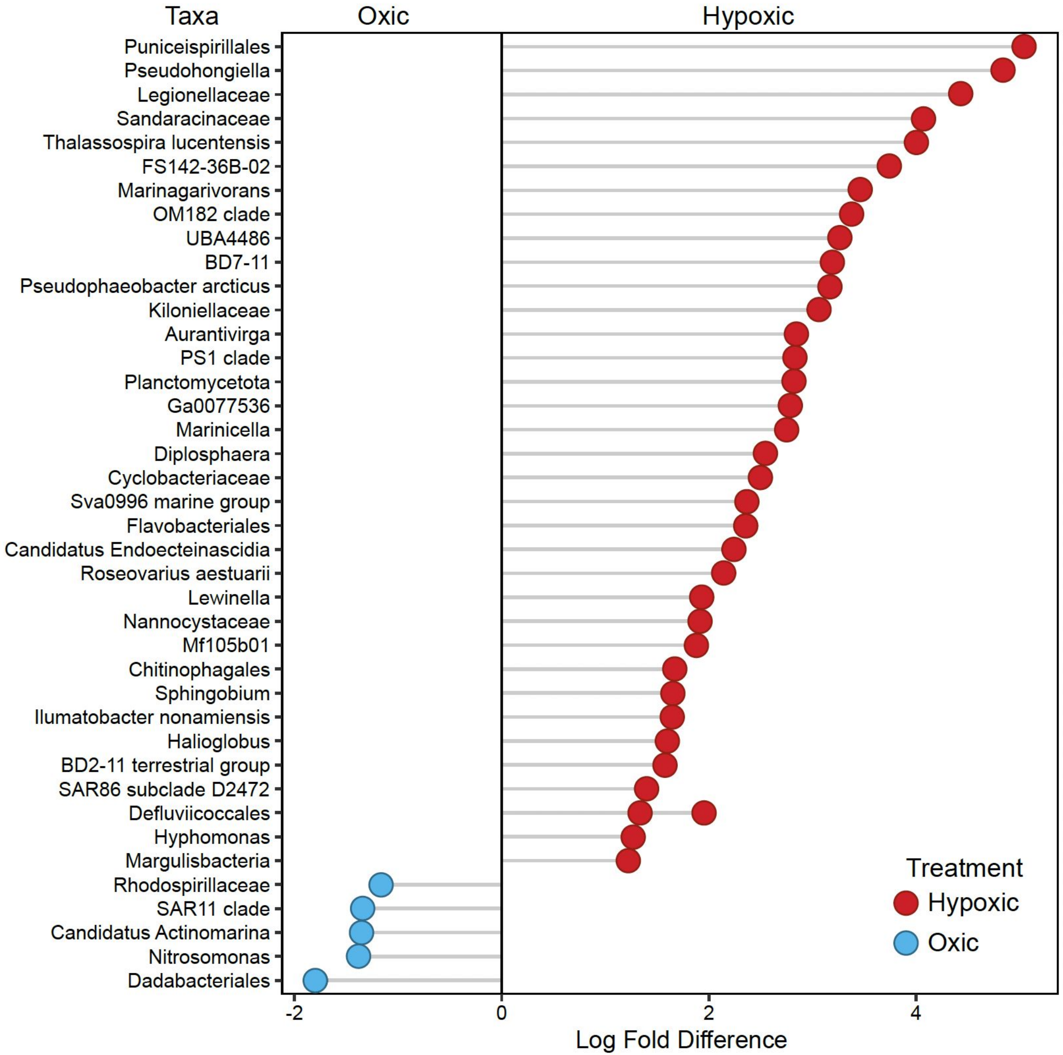
Taxa
Oxic
Hypoxic
Puniceispirillales
Pseudohongiella
Legionellaceae
Sandaracinaceae
Thalassospira lucentensis
FS142-36B-02
Marinagarivorans
OM182 clade
UBA4486
BD7-11
Pseudophaeobacter arcticus
Kiloniellaceae
Aurantivirga
PS1 clade
Planctomycetota
Ga0077536
Marinicella
Diplosphaera
Cyclobacteriaceae
Sva0996 marine group
Flavobacteriales
Candidatus Endoecteinascidia
Roseovarius aestuarii
Lewinella
Nannocystaceae
Mf105b01
Chitinophagales
Sphingobium
Ilumatobacter nonamiensis
Halioglobus
BD2-11 terrestrial group
SAR86 subclade D2472
Defluviicoccales
Hyphomonas
Margulisbacteria
Rhodospirillaceae
SAR11 clade
Candidatus Actinomarina
Nitrosomonas
Dadabacteriales
-2
0
2
4
Log Fold Difference
Treatment
Hypoxic
Oxic
