# Supplementary material for: Oxygen-dependent metabolism of dissolved organic matter in hypoxic marine environments
Source: ISME J. 2026 Aug 26;20(1):wrag220. doi: 10.1093/ismejo/wrag220 (PMC13630563; doi:10.1093/ismejo/wrag220)
Supplement: Supplementary file 1 [file supplementary_material_wrag220.zip › supplemental_figure_table_legends_wrag220.docx]

## Supplementary Figures

**Figure S1** - A) Visualization of methods for separating metabolites from mesocosm experiments into SLRcDOMₘ (n = 1,295), ODDOMₘ (n = 291), and LDOMₘ (n = 511). SLRcDOMₘ molecules were defined as those that increased in relative intensity over time under both hypoxic and oxic conditions. ODDOM molecules were identified as those that either increased or remained stable in abundance under hypoxic conditions but decreased under oxic conditions. LDOM molecules were defined as those that decreased in abundance over time in both hypoxic and oxic treatments. B) Visualization of each DOM category reactivity over time within the mesocosm experiments. The difference between TOC measurements at the end of the experiment is attributed to persistent ODDOM.

**Alternative text:** Two-panel figure illustrating the classification and temporal behavior of dissolved organic matter categories in mesocosm experiments. Panel A shows the criteria used to classify metabolites as SLRcDOMₘ, ODDOMₘ, or LDOMₘ based on changes in relative intensity over time under hypoxic and oxic conditions. SLRcDOMₘ increased under both treatments, ODDOMₘ persisted or increased under hypoxia but decreased under oxic conditions, and LDOMₘ decreased under both treatments. Panel B shows a graph indicating the conceptual reactivity of these DOM categories over time, with the final difference in total organic carbon between hypoxic and oxic treatments attributed to persistent ODDOM.

**Figure S2** - DOM functional group identification of ODDOM and selection of ODDOM for respiration assays. A) Possible chemical structures of top 10 SIRIUS score features (SiriusScore > 300) identified as ODDOMₘ and their shared functional groups/structures. B) Possible chemical structures of CANOPUS identified benzenoid features identified as ODDOMₘ with SIRIUS scores > 100 and their shared functional groups/structures. Based on functional groups/structures shared among ODDOMₘ predictions, four ODDOM compounds were selected for testing in respiration assays. Chemical structures were retrieved from PubChem [59].

**Alternative text:** Two-panel figure showing the identification of functional groups/structures associated with ODDOM and the selection of compounds for respiration assays. Panel A displays possible chemical structures for the 10 highest-scoring SIRIUS features classified as ODDOMₘ, highlighting functional groups/structures shared among the predicted structures such as benzoic acids, benzenes, carboxylic acids, and catechol’s. Panel B shows possible chemical structures of CANOPUS-classified benzenoid ODDOMₘ features with SIRIUS scores greater than 100 and their shared functional groups/structures. Four ODDOM compounds were selected for respiration assays based on functional groups commonly observed among ODDOMₘ predictions.

**Figure S3** - All metabolic pathways for compounds used in respiration experiments identified in KEGG. Not all metabolic/catabolic pathways are shown. All ODDOM require at least one catabolic oxygen-utilizing transformation whereas all LDOM have a metabolic pathway that does not require oxygen prior to cellular respiration. See Table S13 for all enzymes listed.

**Alternative text:** Diagram summarizing KEGG metabolic pathways associated with compounds used in the respiration experiments. ODDOM compounds are shown with catabolic pathways that include at least one oxygen-utilizing transformation before cellular respiration, whereas LDOM compounds are shown with at least one metabolic pathway that can proceed without an oxygen-dependent step prior to respiration. Only representative metabolic and catabolic pathways are displayed.

**Figure S4** - Weighted chemical characteristics of the DOM pool within the hypoxic and oxic mesocosms. A) Temporal trends in chemical characteristics and average values (excluding day 0) are presented as boxplots, with t-test results and corresponding *P* values indicated: AI - aromaticity index, NOSC - nominal oxidation state of carbon, DBE - double bond equivalence, TOC - total organic carbon (µM), MLB - molecular lability boundary, iSD - inverse Simpson diversity. B) Principal Component Analysis (PCA) of chemical characteristic values. Directions indicate the influence of each chemical characteristic on determining the position of each point: OC - oxygen/carbon ratio, HC - hydrogen/carbon ratio.

**Alternative text:** Two-panel figure comparing weighted chemical characteristics of dissolved organic matter in hypoxic and oxic mesocosms. Panel A shows graphs of temporal trends and boxplots of average values, excluding day 0, for aromaticity index, nominal oxidation state of carbon, double bond equivalence, total organic carbon, molecular lability boundary, and inverse Simpson diversity, with t-test results and *P* values indicated. Panel B shows a principal component analysis of chemical characteristics, with vectors indicating the direction and relative influence of each variable, including oxygen-to-carbon, hydrogen-to-carbon ratios, aromaticity index, nominal oxidation state of carbon, and double bond equivalence on sample position in ordination space.

**Figure S5** - All Michaelis-Menten curves from respiration experiments. ODDOM compounds are indicated in red and LDOM compounds are indicated in blue. All rates were fitted to a Michaelis-Menten model and the R² values are reported. See Table S7 for details.

**Alternative text:** Michaelis-Menten curves for all compounds tested in respiration experiments. Four ODDOM compounds are shown in red across eight experiments and four LDOM compounds in blue across seven experiments. Respiration rates are plotted across oxygen concentrations and fitted with Michaelis-Menten models, with the R² values reported for each fit.

**Figure S6** - Relative abundance of photosynthetic lineages through time within the mesocosm experiments.

**Alternative text:** A graph showing the relative abundance of photosynthetic microbial lineages over time in the mesocosm experiments under hypoxic (red) and oxic (blue) conditions.

**Figure S7** - Differential abundance of ASVs identified to their lowest taxonomic level across treatments from day 42 to day 96. The taxa shown differed significantly (*P* < 0.05) between hypoxic and oxic treatments. Positive values indicate taxa that were more abundant in the hypoxic treatment.

**Alternative text:** Plot showing differential abundance of ASVs between hypoxic and oxic treatments from day 42 through day 96, with ASVs labeled at their lowest resolved taxonomic level. Only taxa that differed significantly between treatments (*P* < 0.05) are shown. Positive values indicate greater relative abundance under hypoxic conditions, while negative values indicate greater relative abundance under oxic conditions.

## Supplementary Tables

**Table S1** - Carboy oxygen measurements throughout the 100-day mesocosm experiments.

**Table S2** - Oxygen utilization slope fitting and rate extraction for the 100-day mesocosm experiments.

**Table S3** - Oxygen utilization rates from the 100-day mesocosm experiments and statistical comparisons.

**Table S4** - Curated KEGG database of oxygen-utilizing reactions.

**Table S5** - Methods for all respiration experiments on whole communities and amended compound concentrations for treatments.

**Table S6** - Metadata from respiration experiments.

**Table S7** - Michaelis-Menten curves for each respiration experiment.

**Table S8** - Reported Kₘ and V_max_ for each respiration experiment along with their potential oxygen-utilizing enzyme.

**Table S9** - Nutrient and TOC measurements from the 100-day mesocosm experiments.

**Table S10** - Weighted DOM characteristic values through time in the 100-day mesocosm experiments.

**Table S11** - Model fit to each oxygen utilization curve of each compound (sigmoidal vs exponential).

**Table S12** - Differentially abundant taxa within mesocosm experiments.

**Table S13** - Enzymes shown in Figure S3.
