# Supplementary material for: Oxygen-dependent metabolism of dissolved organic matter in hypoxic marine environments
Source: ISME J. 2026 Aug 26;20(1):wrag220. doi: 10.1093/ismejo/wrag220 (PMC13630563; doi:10.1093/ismejo/wrag220)
Supplement: Supplementary file 1 [file supplementary_material_wrag220.zip › Supplementary_methods_wrag220.docx]

## Supplemental Methods Section (SMS):

1.1 - Mesocosm phytoplankton bloom methods

Chl a was measured in triplicate by filtering 10 mL of seawater onto 25 mm glass fiber filters (Whatman GF/F, Cytiva, Marlborough, MA, USA) and extracting for 24 h at −20°C in 90% acetone. Absorption was measured using a UV-Vis spectrophotometer (Shimadzu, Kyoto, Japan) and calculated as described in Ritchie (Ritchie, 2006).

1.2 - 16S rRNA gene analysis

Processed reads were trimmed at 210 bp (forward) and at 180 bp (reverse). This resulted in 3,332 Amplicon Sequence Variants (ASVs), with 36,842 ± 7,851 reads per sample. ASVs were assigned a taxonomic classification using a trained feature classifier based on a V4-trimmed SILVA gene reference database (v132.2, Quast et al., 2012). Reads assigned to chloroplast lineages were re-classified using a trained feature classifier based on the V4-trimmed chloroplast subset of the PR2 database (v15.5, Guillou et al., 2013). Singleton ASVs were removed from the dataset prior to quantitative analysis.

1.3 - TOC measurements

High-temperature combustion was used to directly measure TOC content. 40 mL samples were collected bi-weekly/weekly and a selected six sampling sets were sent for analysis. Samples were analyzed on a modified Shimadzu TOC-V as described in Halewood et al., 2022 (Halewood et al., 2022). Briefly, 3 mL of sample was acidified with 2 mol L-1 HCl (1.5%) and sparged for 1.5 min with CO2-free gas. Three to five replicates (100 μL) of sample were injected into the combustion tube heated to 680 °C that had CO2-free gas flowing through the system at 168 mL min−1. A magnesium perchlorate trap and copper mesh trap were used to ensure removal of water vapor and halides from the gas line prior to entering a nondispersive infrared detector. The resulting peak area was integrated with Shimadzu chromatographic software.

2.1 - Mass spectrometry methods

The UHPLC separation was performed using a C18 core-shell column (Kinetex, 150 × 2.1 mm, 1.8 µm particle size, 100 A pore size, Phenomenex, Torrance, USA). The mobile phases used were solvent (A) **H_2_O** (LC/MS grade, Fisher Scientific) + 0.1% Formic Acid (FA) and solvent (B) Acetonitrile (LC/MS grade, Fisher Scientific) + 0.1% FA. After the sample injection, a linear gradient method of 10 minutes was used for elution of small molecules, the flow-rate was set to 500 μl min^-1^. The following separation conditions were set: from 0-0.5 min, 5% B, 0.5-8 min from 5 to 50% B, 8-10 min from 50 to 99% B, followed by 3 min washout phase at 99% B and 3 min re-equilibration phase at 5% B. The measurements were conducted in positive mode and the Heated Electrospray Ionization (HESI) parameters included a sheath gas flow rate of 50 AU, auxiliary gas flow rate of 12 AU, and sweep gas flow rate of 1 AU. The spray voltage was set to 3.50 kV and the inlet capillary temperature to 250°C, while the S-lens RF level to 80 V and the auxiliary gas heater temperature to 400°C.

Full MS survey scan acquisition range was set to 150–1,500 m/z with a resolution Rm/z 200 120,000, automatic gain control (AGC) of 1E6, maximum injection time of 100 ms with one micro-scan. MS/MS spectra acquisition was performed in data dependent acquisition (DDA) mode with TopN set to (5), as consequence the five most abundant precursor ions of the survey MS scan were destined to MS/MS fragmentation. The resolution of the MS/MS spectra was set to Rm/z 200 15,000, the AGC target to 5 and the maximum injection time to 200 ms. The isolation window for the precursor ion selection was set to 1.0 m/z and the normalized collision energy was set to a stepped increase from 25 to 35 to 45. MS/MS experiments were triggered at the apex of peaks within 2–15 s from their first occurrence. Dynamic exclusion was set to 5 s. Samples were analyzed in two separate runs on the MS/MS and the runs were analyzed together as replicate samples. MS1 features with peak areas normalized to the total ion chromatogram (i.e., relative abundances) exceeding 3x process blanks were compared among samples. Process blanks were generated in a similar manner using 1 L of water (LC-MS grade) filtered through an Omnipore filter. Assuming similar sample collection and instrumental conditions across all samples presented here, a log2-fold decrease > 1.8 in MS/MS peak area indicated a decrease in the abundance of compounds with time. Molecular spectra were processed via MZmine2 (Pluskal et al., 2010; Schmid et al., 2020), and a quantification feature table (.csv). The generated .mgf files were submitted for Ion-Identity Molecular Networking in the Global Natural Product Social Molecular Networking (GNPS; gnps.ucsd.edu) using similar parameters as outlined in Stephens et al., (2020) (Stephens et al., 2020). The molecular network can be accessed at: <https://gnps.ucsd.edu/ProteoSAFe/status.jsp?task=4abe69939a4749d2b48ea0133f19326c>.

2.2 - DOM annotations

LC-MS/MS data processing was conducted using workflows based on International Chemical Identifiers of known library and analog matches in Classyfire (Djoumbou Feunang et al., 2016). 8,567 features were identified after filtering out blanks and Sterivex controls with a cutoff of 0.3 from the initial 12,018 features (Shah et al., 2023).

2.3 - DOM sorting

DOM was sorted based on the following criteria. LDOM_M_ consisted of compounds decreasing in abundance over time in both treatments (Pearson correlation < -0.7, R² value > 0.7), SLRDOM_M_ consisted of compounds increasing in abundance over time in both treatments (Pearson correlation > 0.7, R² value > 0.7), ODDOM_M_ consisted of compounds 1) increasing in the hypoxic treatment (Pearson correlation > 0.7, R² value > 0.7) and decreasing in the oxic treatment (Pearson correlation < -0.7, R² value > 0.7), or 2) compounds that are increasing in the hypoxic treatment (Pearson correlation > 0.7, R² value > 0.7), non-correlative in the oxic treatment (Pearson correlation > -0.7 and < 0.7, R² < 0.7), Spearman relationship between treatments < 0.7, and not significantly more abundant in the oxic treatment (p > 0.05 and/or LogFoldCh < 0.57), or 3) significantly higher in abundance in the hypoxic treatment (p < 0.05 and/or LogFoldCh > 0.57), significantly decreasing in the oxic treatment (Pearson correlation < -0.7, R² value > 0.7), and a Spearman relationship between treatment < 0.7.

3.1 - Respiration experiment methods

Seawater was filtered through 20 µm nylon mesh and held in the dark for 16 - 24 hours before filtration through a 0.7 µm polycarbonate filter to remove particulate organic matter. Cells were collected on a 0.2 µm Omnipore GV filter and resuspended in ASW without any carbon source. Microbial cells were incubated at 4 °C for a minimum of 24 hours prior to respiration assays to deplete labile intracellular carbon reserves and were operationally defined as starved cells.

3.2 - Respiration rate methods and equations for respiration experiments

Respiration rates were derived from the initial slopes of oxygen utilization, after the temperature of bottles stabilized and before cell growth caused exponential rises in respiration. Linear regression models were fitted to each bottle and only R² ≥ 0.65 were used for O_2_ K_m_ calculations. Experiments with control bottles (containing no cells and only the treatment compound) with an R² ≥ 0.65 were corrected for abiotic reactivity (gentisate and protocatechuate) with dissolved oxygen using the following equation:

$$Rc = Ro-(\frac{Ra}{[O2]a})\cdot[O2]b$$

Where *Rc* = corrected rate, *Ro* = original bottle rate, *Ra* = abiotic reaction rate, *[O2]a* = average oxygen concentration for abiotic reaction, and *[O2]b* = average oxygen concentration for bottle rate. O_2_ K_m_ values were calculated by plotting the inverse rate of oxygen utilization with the inverse average oxygen concentration on a Lineweaver-Burk plot.

Oxygen concentration vs time was fitted to two models; the Single-phase Exponential Model indicating monophasic kinetics:

$$f(x)=A-B\cdot exp(-r\cdot x)$$

and the Sigmoidal Gompertz Decay Model indicating biphasic kinetics:

$$f(x)=0+(d-0)exp(-exp(b(log(x)-log(e))))$$
